# Traditional, Therapeutic Uses and Phytochemistry of Terrestrial European Orchids and Implications for Conservation

**DOI:** 10.3390/plants12020257

**Published:** 2023-01-05

**Authors:** Miriam Bazzicalupo, Jacopo Calevo, Antonella Smeriglio, Laura Cornara

**Affiliations:** 1 Department of Earth, Environment and Life Sciences (DISTAV), University of Genova, 16132 Genova, Italy; 2 CREA—Research Centre for Vegetable and Ornamental Crops, Council for Agricultural Research and Economics, 18038 Sanremo, Italy; 3 Department of Ecosystem Stewardship, Jodrell Laboratory, Royal Botanic Gardens, KEW, Richmond, Surrey TW9 3DS, UK; 4 School of Molecular and Life Sciences, Curtin University, Perth, WA 6102, Australia; 5 Department of Chemical, Biological, Pharmaceutical and Environmental Science, University of Messina, 98166 Messina, Italy

**Keywords:** ethnobotany, Orchidaceae, biological properties, threatened species

## Abstract

The Orchidaceae family accounts for about 28,000 species, and most of them are mentioned in the folk medicine of nations around the world. The use of terrestrial orchids in European and Mediterranean regions has been reported since ancient times, but little information is available on their medicinal properties, as well as on their phytochemicals and biological activities. However, plant collection for human use is still listed as one of the main threats for terrestrial orchids, alongside other menacing factors such as wrong habitat management and disturbance to symbionts, such as pollinators and mycorrhizal fungi. Therefore, the primary aim of this review was to resume and discuss available information regarding the past and current popular uses of European orchids. We then grouped phytochemical data to evaluate the presence of bioactive compounds of pharmacological relevance, and we discussed whether these could support the therapeutic employment of the different organs. Finally, we briefly debated the sustainability of orchid utilizations, considering the different threatening factors and conservation actions including plant propagation methods.

## 1. Introduction

Family Orchidaceae, with approximately 28,000 species distributed worldwide except in the poles and deserts, is considered one of the most fascinating and diverse group of plants among angiosperms [1,2]. In fact, orchids show a wide variety of life forms, habitat preferences, reproductive strategies, sizes, colours, and scents, characteristics that have placed them at the centre of the attention of many researchers and passionate people [2,3,4]. Despite their evolutionary success, orchids are among the most endangered plants in the world [2,5], mainly because of their strict dependency on interactions with pollinators and mycorrhizal fungi for spreading and persistence, which leads to species being negatively affected by climate change, use of pesticides, anthropogenic pressure, human harvesting, etc. According to recent estimates, hundreds of species are threatened, with terrestrial orchids particularly represented in the IUCN Red list [6,7,8]. All members of Orchidaceae have therefore been included in Appendix II or higher of the Convention on International Trade in Endangered Species, CITES. Orchids are indeed highly represented in the commerce, being traded legally or not for their ornamental value, or as source of components for cosmeceuticals and medicine, or as food [9,10]. Orchids are also known in the folk tradition of many nations around the world [11,12,13]. Since ancient times, orchids have been used as nourishment and have also been employed in medicinal preparations. The first descriptions of orchids and their therapeutic utilizations have been found in China since 2800 B.C. [14], while in ancient Ayurvedic preparation Ashtavarga, four terrestrial orchids are included (*Herminium edgeworthii* (Hook.f. ex Collett) X.H. Jin, Schuit., Raskoti and Lu Q. Huang; *Habenaria intermedia* D. Don, *Crepidium acuminatum* (D. Don) Szlach., and *Malaxis muscifera* (Lindl.) Kuntze) [12,13,15]. It is reported that orchids were used for the treatment of diseases and ailments such as tuberculosis, paralysis, gastrointestinal problems, chest pains, syphilis, arthritis, cholera, cancers, piles, boils, muscular pains, menstrual disorders, diarrhoea, leucorrhoea, hepatitis, spermatorrhea, rheumatism, wounds, sores, and others [11,12,13,15]. Therefore, it has been stated that orchids possess a high medicinal potential as a source of drugs [11,13,15,16,17].

Orchids are known to produce secondary metabolites of physiological, ecological, and pharmacological relevance [13,18,19,20,21,22,23]: among these, compounds such as stilbenes, dihydrostilbenoids, phenanthrenes, alkaloids, terpenes, flavonoids, anthocyanins, and phenolic acids have been found [12,13,23]. However, notwithstanding the number of species in the family, relatively few studies were dedicated to orchid phytochemistry and biological activities, and many traditional uses remain unvalidated [12,15]; this is particularly evident for European species, although it is known that Europe has a well-established traditional use of these plants [12,13,24,25,26,27,28]. One of the first reporting orchids in medicine was the Greek Theophrastus (c.372–288 B.C.), who named orchids on the base of the similarity of their tubers with male testicles (“όρχεις”) and suggested their use as an aphrodisiac [29]. Pliny the Elder (23–79 A.C.) stated that sexual desire could be increased by consuming the harder/bigger bulb, while it could be repressed by consuming the softer/smaller one. Furthermore, according to Pliny, underground portions of orchids could be employed to cure mouth sores or to clear the phlegm from the chest [13,30]. The belief that the orchids could influence the sexual sphere has been then proposed for centuries: Petronius, in his *Satyricon* (1st century A.C.), described the consumption of orchids among prostitutes; in the texts of Dioscorides and Galen, these plants were cited following the *Doctrine of Signatures,* as also reported by Paracelsus (1493–1551) and Linnaeus (1707–1778) [31].

The European Union, in addition to applying the CITES, started establishing the Habitat Directive and the Natura 2000 Network about thirty years ago, purportedly created for the protection of habitats and animal and plant species. Various orchids are also included in national and regional red lists, thus the collection of threatened species is now regulated or forbidden [5,27].

European orchids are often cited in papers on ethnobotanical knowledge, but an updated review fully dedicated to the species from the Old Continent is still missing. Therefore, the purpose of the present review is to summarize ethnobotanical uses reported in the folk tradition, and to evaluate whether these uses are sustainable, are still practiced and can influence the persistence of species. Furthermore, we collected available phytochemical information and discussed biological properties of these plants, which could eventually justify ethnobotanical uses. We finally debated the sustainability of collection, with implications for species conservation.

Note that all the species are hereby cited with their current accepted names according to IPNI (https://www.ipni.org/, accessed by 14 December 2022) and POWO (https://powo.science.kew.org, accessed by 14 December 2022). Any synonymies and infraspecific taxa are included under the category of the relative accepted species *sensu lato.* Information concerning conservation status is reported here only for the species mentioned in ethnobotanical reports and according to the IUCN Red List of Threatened Species (https://www.iucnredlist.org/, accessed by 14 December 2022).

## 2. Ethnobotanical Uses

In our study, we obtained information on ethnobotanical uses for a total of 62 native species. Traditional knowledge on orchids was found in Turkey, Serbia, Bosnia-Herzegovina, Italy, Greece, Great Britain, Hungary, Macedonia, Albania and South Kosovo, European Russia, Central Europe, and Spain. All the cited taxa are geophytes, of which 58 are tuberous and four are rhizomatous. Overall, the hypogean apparatus was the most cited portion. Harvesting of tubers for Salep (see below) was considered separately from the collection for alimentary/medicinal home consumption, as well as the ritual use.

Multiple uses were found for 40 species. For 47 taxa, the hypogean portion was cited for Salep production (75.80% of the total investigated species), while in 41 cases it was consumed as medicinal food (66.13%); the tuber was utilized in rituals only in five cases (8.06%). Other organs were less cited: leaves/flowering stem were used as medicinal in eight cases (12.90%), and in rituals in five cases (8.06%). In 16 cases, the flowering stem was used as decorative (25.80%), while in four cases the whole plant was harvested and moved to kitchen gardens as ornamental (6.45%).

Overall, the most cited species are *Anacamptis morio* (L.) R.M. Bateman, Pridgeon and M.W. Chase, and *Orchis mascula* (L.) L.

The complete list of all the ethnobotanical information that we found by examining original papers, books and reviews is provided in Table 1. Part of the source bibliographic material that we collected was still untranslated, being available only in the authors’ original language (especially for Italy).

### 2.1. Current Uses

**Figure 1:**
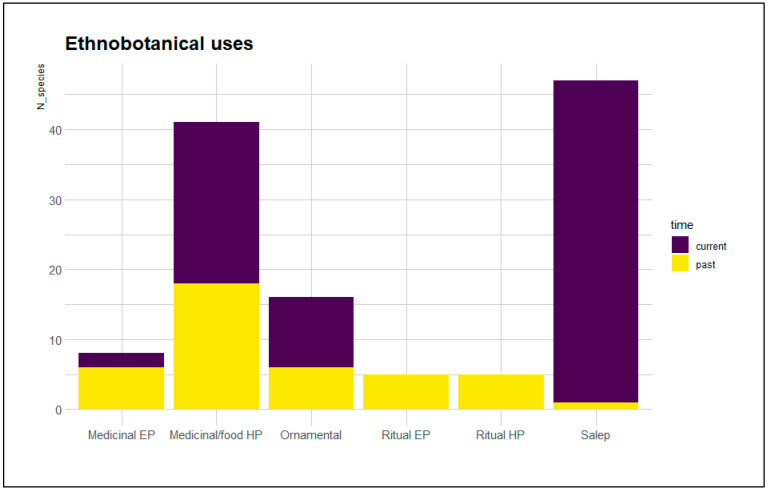
Comparison between past and current uses of European orchids. EP: epigean portion; HP: hypogean portion.

We considered as current the uses reported in relative ethnobotanical investigations carried out and published after the year 2012 (past ten years). We found that the folk uses continue today for 54 species. All the tuberous orchids cited for Salep are currently harvested, while the consumption of tuber as food/medicinal ingredient other than Salep continues for 23 species. In these cases, the highest number of citations was found for Turkey, Bulgaria, Greece, and Serbia. Only *Dactylorhiza euxina* in Turkey and *Gymnadenia rhellicani* in Italy are harvested today at the above-ground level for medicinal purposes. We found that 10 species are still mentioned for ornamental aims: in these cases, cited countries were mainly Italy and Turkey. Finally, ritual uses are no longer reported. A graphical comparison between current and past utilizations of the different portions is shown in Figure 1.

### 2.2. Orchids and Salep

The mixture called Salep, for which the majority of our studied species were mentioned, is the most famous folk preparation that includes orchids. Given its ethnic importance and the associated concerns it creates for orchid conservation, much of the literature has already been dedicated to this product. For thousands of years, as reported for other species from Indian region and Middle East, terrestrial orchids have been harvested to ground their tubers and produce Salep powder [13,24,25,26,27,32,66,139,162,163,164]. Salep is also the name attributed to dried orchid tubers. This preparation originated in the Middle East in ancient times but had returned to be famous in the Europe of Renaissance after the publication of *Gerard’s Herbal* in 1633 [13]. We found 46 tuberous and one rhizomatous species mentioned for Salep, but Tamer et al. [162] stated that 90 different taxa are overall harvested for this aim: tubers belong mainly to genera *Orchis*, *Anacamptis*, and *Ophrys*, but also *Dactylorhiza*, *Neotinea*, *Himantoglossum*, *Serapias*, and *Platanthera* are cited. As highlighted in Table 1 and as confirmed by various authors, the consumption of Salep is not only intended for alimentary use, but it is highly considered for the treatment of coughs, fever, diarrhoea, intestinal problems, asthenia, and to increase appetite and sexual desire. The substance is served as the homonymous hot drink Salep or it is an ingredient for sweets, being especially employed in the derivate ice-cream called “Salepi dondurma” in Turkey and “Kainaki” in Greece [24,32,139,162,165].

### 2.3. Uses of Orchid Below-Ground Portions

**Figure 2:**
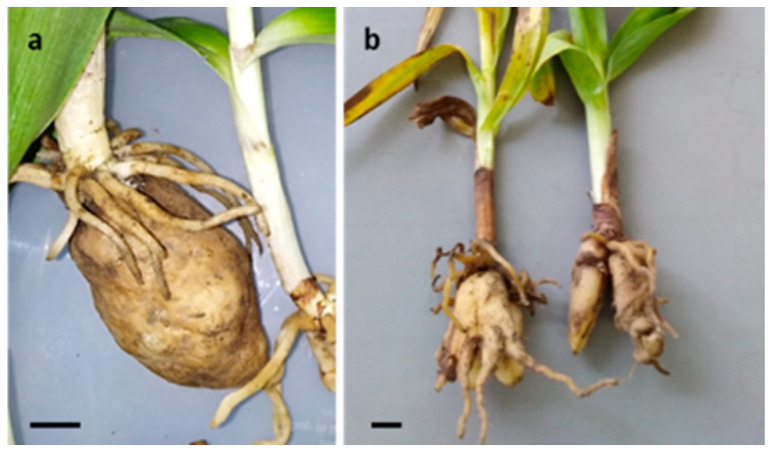
Bar = 1 cm. (**a**) The large tuber of the giant orchid (*Himantoglossum robertianum*); (**b**) palmate tubers from *Dactylorhiza sambucina*.

In Europe and especially in the Mediterranean Area, orchid tubers are not only harvested for Salep preparation, but they are mentioned for home consumption as medicine or food. During the Renaissance, Mattioli [51] had already indicated tubers from *A. morio* as source of nourishment for the inhabitants of Trentino and Ladinia Dolomitica (Northern Italy). Orchids were reported to be eaten in several Italian regions to recover some nutrients, especially during wars and famine. Mattirolo [52], in this context, listed eight orchid species (see Table 1) in his book *Phytoalimurgia Pedemontana* (1918), with which he aimed to help rural Italian people to survive the food crisis caused by the First World War. The large tubers of *H. robertianum* (Figure 2a) were roasted and eaten in Sicily [109,110]. Similar uses for this plant, together with *O. apifera*, *N. ustulata*, and other unspecified *Ophrys* and *Dactylorhiza* species, were reported for Sardinia [82,108] where tubers were consumed in the traditional preparation “Kasùgottu” (literally, “cooked cheese”). For this region, Ballero and Fresu [166] indicated tubers from *Orchis s.l.* for the cure of childhood diarrhoea. For Italy, Guarrera [82] reported alimentary and medicinal use for *Dactylorhiza*, *Ophrys*, and *Orchis*. Tubers of *O. anthropophora* were reported as food source and to cure phthisis, pectoral ailments, and hemoptysis [97,140]. In Northern Europe, Fousch (1549), and then Turner (1568), Langham (1579), and Parkinson (1640), indicated orchid tubers as medicinal resource to treat skin and gastrointestinal problems, and as anti-pyretic, anti-septic, anti-consumption, anti-diarrhoea, and to enhance sexual activity [14].

In South-Eastern Europe (Turkey, Serbia, Bulgaria, Albania, South Kosovo and the Balkans, Hungary) tubers are still mentioned for medicinal purposes. Orchid species are prepared in decoction/infusion and taken as panacea to treat several conditions, such as cough, cold, inflammation, infections/abscess, wounds, boils and skin diseases, respiratory and gastrointestinal disorders; furthermore, uses as tonic, for mental wellness and aphrodisiac have been reported. Similarly, hypogean portions of rhizomatous species were harvested as herbal remedies: *Cypripedium calceolus*, *Epipactis helleborine*, *Neottia ovata* and *Spiranthes spiralis* were employed to obtain sedative and neuroactive effects, or to treat rheumatisms, skin and mucosal problems, or as aphrodisiac [11,13,117].

Ritual uses of orchid tubers have also been cited, always referring to the sexual or emotional sphere. Decorticated tubers from *Orchis sp. pl*. were reported to be aphrodisiac in the folk tradition of Friuli–Venezia–Giulia (Italy) and were employed to prepare “love decoctions” [167]. In Great Britain, a similar use was indicated for taxa that according to successive botanical investigations could be possibly identified with *O. mascula*, *D. purpurella* (T. Stephenson and T.A. Stephenson) Soó, or *D. maculata* [118]. In Trentino and Ladinia Dolomitica (Italy), male farmers had to look for orchids with palmate tubers (Figure 2b) and the girls those with testicular ones to eat them as a mutual demonstration of sexual sympathy [50]. In Northern Piedmont (Italy) a decoction with *Nigritella*’s tubers (syn = *Gymnadenia sp.*) was secretly administered to the members of a couple: if during cooking the tubers remained close together it meant concord, vice versa discord. In Abruzzo (Central Italy), the tubers of *G. conopsea* or *O. purpurea* were used in ceremonies to reunite or separate couples [82,151]; for the same reason, *D. maculata* was collected in Sardinia [82].

### 2.4. Uses of Orchid Aerial Parts or Whole Plant

In the European traditional knowledge, while tubers are widely mentioned, few reports on the utilization of other portions of orchids are available. Both food and medicinal, ritual, and ornamental uses are known. In Turkey, a culinary recipe that mentions the use of leaves of *O. mascula* subsp. *mascula* cooked with onion and eggs was found ([66] and references therein). In Sardinia (Italy), leaves of *E. helleborine* were used for the treatment of wounds [97], while those of *D. euxina* are still cited for this purpose in Turkey [74]. In North-Eastern Italy, dry leaves of *Platanthera bifolia* were cooked in water–vinegar and then directly applied on the skin for the cure of rheumatism and as anti-neuralgic [155]; in Dorset (UK), aerial parts of *P. chlorantha* were included in an ointment to be applied on ulcers [118]. Leaves and flowers of *D. sambucina* were harvested in Liguria (Praglia; Genoa, Italy) to prepare a decoction against coughs [92]. In the 1577, Mattioli [51] reported for Northern Italy the use of flowers from genus *Gymnadenia* (*G. nigra*, *G. rhellicani* and related, *G. odoratissima*, *G. conopsea*) in infusion to produce digestive decoctions or distillates. For alpine regions of Northern Italy, the good-smelling inflorescence of *G. rhellicani* is one of the most mentioned: it is reported in various culinary recipes including liquors, as a herbal remedy for cold and respiratory diseases, or for increasing sexual desire [82,101,102,103,104]. Furthermore, shepherds from Aosta Valley had stated that if the cows ate too many *G. rhellicani* flowers, the fontina cheese became bitter [104]. Concerning other uses of orchid epigean portions, the inflorescence of *D. maculata* was considered psychoactive and was used by the sorcerer “Marendìn” to hypnotize women in Northern Italy during the XVII century [80]. The flowering stem of *Anacamptis papilionacea* was employed in spells and evil eye in Sicily, Italy [61]. In Liguria (Vara Valley—Italy), in early May (“*au primmu su de Mazzu* […]”) children were rubbed with the inflorescence of *O. mascula* and other orchids to protect them from the bites of snakes and other animals [145]. In Aosta Valley, Italy, flowers of *G. rhellicani* were gifted by boys to girls they were interested in [104]; a similar approach was reported for *Ophrys scolopax* in Spain by González et al. [128]. Several species are mentioned for being collected as ornamentals at the stem level or as entire plants because of their beauty and/or scents: both in Italy and Turkey, this practice was particularly widespread. Among the reports, many different *Ophrys* are cited, i.e., *O. tenthredinifera* and *O. sphegodes* (see Table 1), together with *O. argolica* in Turkey [25] that is listed as Vulnerable, and *O. speculum* in Liguria [99], nowadays rarely observed in this region. The harvest of the rare *D. insularis* in Sardinia [53] also deserves a special mention.

Finally, skincare products industrially made with extracts from flowers of *Orchis mascula, D. maculata*, or *A. morio* are commercially available [9].

## 3. Phytochemical Information and Pharmacology of European Orchids

We found phytochemical information for 88 orchids diffused in European territories, of which 85 were selected based on the presence of known bioactive compounds; details on infraspecific taxa were also included. Table 2 provides a partial database of literature reporting compounds detected, and relative evaluations of biological properties, if present. As highlighted in the table, most of the available information consists in metadata collected from articles written for very different aims, namely works on chemical ecology for the evaluation of plant–pollinator interactions, or phytochemical analyses on the presence of specific classes of compounds compared in different taxa, even in a phylogenetic key (i.e., Strack et al. [168]). From a phytochemical point of view, the flowers/inflorescence are therefore the portions on which more information is reported, with more than 70 species being investigated; the most studied are *O. mascula s.l.*, *A. coriophora s.l.*, *G. conopsea*, *H. robertianum*, *Ophrys sphegodes* complex, and *P. bifolia.* Specific groups of chemical components of the leaves were found for 33 species, especially thanks to the work of Williams [169], van Damne and colleagues [170,171], and Balzarini [172]; the most cited orchids are *E. atrorubens* (Hoffm.) Besser, *E. helleborine*, *N. ovata*, and *A. papilionacea*. Phytochemical details for the hypogean apparatus (tuber/rhizome) are available for a total of 17 species, especially deriving from investigations on phytoalexin compounds and their production in response to external stimuli (see below). Many authors focused on the biochemicals and biological properties of extracts from tuber of *D. viridis* (L.) R.M. Bateman, Pridgeon and M.W. Chase, and *G. conopsea* (two orchids presenting a wide global distribution); the latter taxon has been already the subject of a review [16] (these results were not included in the table). These two species in particular (and a few others as well, i.e., *Goodyera repens* (L.) R.Br., *Orchis mascula*), being also present in the folk traditions and pharmacopoeia of other extra-EU countries, have therefore been studied by non-European working groups. Finally, starch [173], ash, sugar, sucrose [13], and glucomannan [38,84] were detected in orchid tubers.

### 3.1. Bioactive Compounds, Tissue Distribution and Main Biological Properties

Among secondary metabolites, polyphenols and derivatives are largely studied, and the various polyphenol subgroups have been frequently reported in the investigated species. These subgroups consist in compounds such as flavonoids (including flavanones, anthocyanins, flavonols) and phenolic acids (including caffeic acid or chlorogenic acid). Among the health-promoting activities of polyphenols, antioxidant, cytotoxic, anti-inflammatory, antihypertensive, skin-preserving, and anti-diabetic properties have been evaluated by both *in vitro* and *in vivo* assays [240].

The extensive investigation performed by Strack et al. [168] and Uphoff [177] on the anthocyanin content and relative patterns of abundance allowed to obtain information for many European orchids. Interestingly, the relative content of identified pigments (chrysanthemin, cyanin, seranin, orchicyanin I, ophrysanin, serapianin, orchicyanin II, mecocyanin, and epipactin), and unidentified ones from this water-soluble class of flavonoids was found to be highly variable but genus specific. According to these authors, Arditti and Fisch [241] and Uphoff [242], the mixture of acylated and non-acylated anthocyanins underlies the great variability of orchid flower colours, with orchicyanin I recognized as one of the key compounds responsible for intensive flower pigmentation. With about 600 identified compounds, anthocyanins have strong antioxidant properties and a validated defensive role for plants against biotic or abiotic stressors. These pigments are also active in delaying organ senescence, therefore contributing to the prolongation of tissue survival and increasing reproductive success. Patterns of anthocyanins also have a well-documented role in pollinator attraction [243,244]; i.e., in the case of *Ophrys* species, chrysanthemin and ophrysanin have been traced to the darker pigmentation of the labellum [168], which is typically one of the traits helping the flower in mimicking the female of the insect by which it is pollinated. Intriguingly, Vignolini and colleagues [218] found that the appearance of the speculum in *O. speculum* depends not only on the morphology of the surface cell layer, but also on the concentrated localization of cyanidin pigments. Colourful pigmentation (which is based on anthocyanins) has also been linked to the increased attention of people in regards of plants [4].

In orchid species, compounds with a recognized role as phytoalexins have been found (see Table 2; [13,16,178,179,194,227,245,246,247]). These secondary metabolites are a super-group of compounds (such as flavonoids, terpenoids, coumarins, stilbenoids/phenanthrenes and derivatives, glycosteroids and alkaloids). They are categorized as phytoalexins if their production starts in response to microbial attacks [248], playing a key role in the resistance against groups of microorganisms. For instance, they are known for exerting antibacterial [17] and fungistatic activity ([13] and references therein, [227] and references therein). Among phenanthrenes and derivatives recognized as phytoalexins, hircinol, militarine, loroglossol, and orchinol are the most common molecules recorded [13,249]. Studies on orchid phytoalexins have been conducted, especially on the hypogean portions (i.e., *Orchis mascula*, *O. militaris*, *H. robertianum*). However, these compounds, such as loroglossin in *A. papilionacea* [179], were also reported in flowers and leaves. Phytoalexins such as loroglossol and hircinol recently re-isolated from the tuber of *H. robertianum* by Badalamenti et al. [17], showed *in vitro* antioxidant and immuno-stimulatory effects, together with anti-microbic and anti-cancer activities. 4-hydroxybenzyl alcohol (or *p*-hydroxybenzyl alcohol) is another well-known molecule that has been detected in different orchid tissues and species (see Table 2), for example, in the tuber of *A. coriophora* ([13] and references therein) or in the flowers of *D. maculata* [184]. This compound has interesting biological activities as assessed by both *in vitro* and *in vivo* tests, such as: effects on the central nervous system (sedative, hypnotic, sleep-promotion properties [250], neuroprotective and anti-Parkinson activity [251], antioxidant, antimicrobial, and skin preserving properties, including anti-tyrosinase activity [13,252]).

Alkaloids were found in different portions of several orchids, including leaves and flowers (Table 2). These molecules, in addition to being in some cases recognized as phytoalexins [248], are well-known for their activity on the animal nervous system. Alkaloids were detected in species like *C. longifolia*, *Goodyera repens* and *E. helleborine* [13,182,204]. In this latter orchid, oxycodone and other morphinan/indole derivatives were also found in the flower nectar by Jakubska et al. [205], which proposed that these molecules could be at the basis of the sluggish effect and the disorientation of visiting insects.

Phytochemicals detected in the flowers/inflorescence (whose presence has been mainly collected from anthecology articles) belong to several classes: apart from polar compounds/less volatile ones like polyphenols [176,184,253], saturated and unsaturated hydrocarbons, fatty acids and derivatives, and ketones are frequently found and proposed to contribute to pollinator attraction or herbivory avoidance ([175] and references therein); the same role has been hypothesized for other classes such as aldehydes, alcohols, esters, coumarins, or terpenes, which are well-known as fragrant compounds. Among these, coumarin and terpenes are important metabolites that possess various physiological, ecological, and therapeutic functions. For example, orchid-derived compounds belonging to diterpenoids, sesquiterpenoids, and triterpenoids have shown interesting antiviral activities, including anti-SARS-CoV-2 properties due to the inhibitory competition on 3CL viral protease. Other compounds such as eugenol and methyl–eugenol are recognized as spicy and have several biological properties, among which are anaesthetic and hepatotoxic [13].

### 3.2. Causes of Biochemical Variations

Some species have been chemically analysed in several studies, but the guilds of compounds were found to be very variable (see Table 2). This may depend on several aspects, including the extraction methods and the analytical tools chosen [20,254,255]. For instance, more polar compounds such as polyphenols are less volatiles and therefore the use of protic solvents (such as methanol, diethyl ether or water–ethanol) results in substantially different chemical characterizations in respect to dynamic headspace sorption methods (i.e., [176,185,192]). Individual situations also contribute to changing metabolic processes and thus varying phytochemical profiles. It is known that the concentration of antioxidants such as polyphenols or carotenoids can change depending on plant physiological/phenological status ([256] and references therein). For example, Maleva et al. [237] found that leaves from plants belonging to disturbed habitats showed an increased content of flavonoids. The sources of variability in orchid floral colour and scent have been already reviewed by Dormont et al. [257]. Concerning the content of anthocyanins in particular, changes have been recorded in plants collected in different populations [168] or in individuals facing nutrient deficiency ([243] and references therein). Differences in alkaloid content between plants from diverse countries have been noted ([13] and references therein). Chemical patterns can also differ due to random genetic drift [233]; some species were found to be phylogenetically similar but chemically distant [258], showing significant differences even between subspecies. Ayasse et al. [259] demonstrated that odour variation between plants of *Ophrys sphegodes* favoured cross pollination; furthermore, Schiestl and Ayasse [260] observed that pollinated flowers increased the production of the repellent farnesyl hexanoate. In support to these reports, Dormont et al. [261] confirmed that factors such as habitat characteristics, flower age, pollination, circadian rhythm, herbivory, and inflorescence morphology are responsible for chemical variations in *Orchis mascula.* Finally, as previously mentioned, the production of phytoalexins increases in tissues under microbial colonization. In this context, it is still to define whether the content of these secondary metabolites is dependent on the plant, or on hosts such as mycorrhizal fungi [13,262,263]. The relative abundance of the biochemical components should therefore be considered rather as a “snapshot” of the species’ phytochemistry in response to a given environmental (physiological or ecological) situation.

### 3.3. Validation of Traditional Uses

When comparing the data on portions used as herbal remedies and the available scientific literature on phytochemistry/biological activities, only a few direct matches can be found. In the following cases, however, only a partial explanation that usually stops at *in vitro* evaluations is available.

*Dactylorhiza romana* subsp. *georgica:* Tuber was cited for the cure of cough. Kotiloğlu et al. [190] and Bozkir et al. [191] recently analysed different extracts, including ethanol extracts from both dried and fresh tuber. Polyphenols and flavonoids were detected. Among constituents, *p*-hydroxybenzoic acid, kaempferol, rosmarinic acid, and caffeic acid were present. In both these studies, extracts demonstrated antioxidant and antimicrobial activities.

*Gymnadenia conopsea*: In Europe, the tuber was cited for the treatment of lung diseases [49]. As mentioned, this species is widespread; thus, other available reports on its medicinal use are originally from Asia, as well as scientific studies on its phytochemistry/biological activities. Compounds such as gymconopins, gymnosides, dactylorhins, bulbocodins, arundinins, batatasin, dactylose A-B, coelovirins A-E, loroglossin militarine, coumaric acids, 4-hydroxybenzyl alcohol, vanillic acid, syringol, eugenol, gastrodin, arctigenin, lappaol, quercitin-3, and 7-di-O-β-D-glucopyranoside have been detected thanks to the work of several authors ([16] and references therein). Among the biological properties tested, antioxidant, antimicrobial, anti-viral, antianaphylaxis, immunoregulatory, sedative, and hypnotic activities have been reported.

*Himantoglossum robertianum*: The tuber was prepared in infusion as medicinal tea and was cited for the cure of coughs. Badalamenti et al. [17], as mentioned above, worked on two phenanthrenes, loroglossol and hircinol, isolated from the tuber. The compounds exhibited *in vitro* antioxidant and immune–stimulatory activity, increasing the activity of superoxide dismutase (SOD), catalase (CAT), and glutathione S-transferase (GST) in polymorphonuclear leukocytes (PMN); they also have antimicrobial properties as demonstrated by tests with *Escherichia coli* and *Staphylococcus aureus*; they have also anti-proliferative effect on gastric tumour cell lines by induction of apoptotic effect. However, the importance of other components of the plant complex in the entire traditional preparation cannot be excluded.

As mentioned above, glucomannan, ash, mucilage, water, and starch have been detected in orchid hypogean portions, therefore justifying their alimentary consumption, and indirectly, that of Salep. However, in this case, some authors in the XIX century already argued that its nutritive potential was overestimated ([13] and references therein). No scientific evidence has been found demonstrating the claimed beneficial and aphrodisiac activities of Salep or constitutive portions of the species employed. Some of the secondary metabolites from orchid tubers (i.e., polyphenols/flavonoids) have well-documented biological properties ([240] and references therein) but have no confirmed role in Salep. It should also be remembered that orchid tubers are strongly processed before pulverisation [264] and that spices such as cinnamon or ginger are added as flavours [13].

Some of the traditional uses cited can be at least partially explained by the available literature on phytochemicals. Compounds such as *p*-hydroxybenzyl alcohol (see above) can underline uses of tubers as anti-inflammatory or for the treatment of skin/gastrointestinal problems (i.e., *A. coriophora* or *A.morio*); the same can be hypothesized for antimicrobial molecules such as orchinol, loroglossol, militarine, or orchinol found in different species (i.e., *Orchis spp.*, *Anacamptis spp.*, *Himantoglossum spp.*, *Dactylorhiza spp.*, *Gymnadenia spp.*, *N. ovata*). The presence of *p*-hydroxybenzyl alcohol, known as neuroactive and sedative, could be also at the basis of the alleged psychoactive properties of *A. pyramidalis* tuber or of *D. maculata* flowers (see Table 1). The tuber of *D. osmanica* was used to cure coughs, inflammation, ulcers, and skin boils: Kiziltas et al. [188], while investigating the tuber extract to evaluate other biological properties, found that fumaric acid, *p*-coumaric acid, rosmarinic acid, and vanillic acid were present. A contribution of these compounds to the beneficial effect of *D. osmanica*’s tuber use cannot be excluded. Leaves and flowers of *D. sambucina* were prepared in infusion and cited for cough treatment: these portions were chemically investigated by Pagani [176], who listed health-promoting compounds such as coumarin, quercetin derivatives, or chlorogenic acid. In this species, water-soluble antioxidant anthocyanins such as cyanin, seranin, ophrysanin, orchicyanin II were recognized by Strack et al. [168]; quoted antibacterial terpenoids such as caryophyllene were found in the floral scent [192]. Leaves of *E. helleborine,* used to cure wounds, showed the presence of the antioxidant, antimicrobial, and wound healer quercetin [169]. Furthermore, alkaloids were detected in this species by Lüning [204].

Finally, leaves/aerial parts of both *P. bifolia* and *P. chlorantha* were employed as herbal remedies, to cure rheumatism, neuralgias, and skin ulcers, respectively. In the case of *P. bifolia,* well-known flavonoids like quercetin and kaempferol were detected in these tissues by Williams [169]; the presence of phenolic compounds, although variable between polluted and unpolluted sites, was confirmed by Maleva et al. [237].

Finally, there are species whose selected portions have been analysed phytochemically and for testing biological activities not reported in the ethnobotanical literature.

*Anacamptis coriophora* subsp. *fragrans:* Essential oil isolated from the inflorescence bearing mature seeds was characterized by El Mokni et al. [174]. Constituents were mainly methyl-(E)-p-methoxycinnamate, 13-heptadecyn-1-ol, 2,5-dimethoxybenzyl alcohol, 4-(1,1,3,3-tetramethylbutyl)-phenol; 7,9-di-tert-butyl-1-oxaspiro (4,5) deca-6,9-diene-2,8-dione; 10-dodecenol, *p*-cresol, methyl (Z) p-methoxycinnamate, 2-dodecenal, and methyl cinnamate. Though the EO showed a weak antioxidant activity, anti-proliferative effect on carcinoma cells BxPC3 and human ovarian cancer cells OV2008 was observed.

*Anacamptis pyramidalis*: Mahomoodally et al. [181] analysed water and ethanol tuber extracts, which showed the presence of flavonoids, gastrodin/dihydroxybenzoic acid/caffeic acid/acacetin derivatives, parishin, and citric acid. Extracts exhibited high antioxidant activity and inhibitory potential against tyrosinase, *α*-amilase, and *α*-glucosidase.

*Himantoglossum robertianum:* Hydroalcoholic flower extract was phytochemically characterized: among the different compounds, authors found scopoletin, kaempferol-3-O-rutinoside, caffeic acid, chlorogenic acid, epicatechin, roifolin, protocatecuic acid, vitexin, isovitexin, coumaric acid, catechin, and apigenin. The extract exhibited antioxidant and skin-preserving properties. Inhibitory activity against skin matrix-degrading enzymes (elastase, collagenase) was evidenced, together with improvement of HaCat keratinocytes viability after treatment with H_2_O_2_, and improvement of cell migration rate [213].

*Dactylorhiza romana* subsp. *georgica*: Antioxidant and antimicrobial activities of the tuber from this species have been already cited above. Kotiloğlu et al. [190] and Bozkir et al. [191] also observed antidiabetic properties by evaluating *α*- amylase and *α*-glucosidase inhibitory activity during *in vitro* assays.

*Dactylorhiza osmanica*: This species and its phytochemicals have been already mentioned above. Kiziltas et al. [188] also found that both tuber and flowering stem extracts have anti-Alzheimer and anti-diabetes properties, as evaluated by *in vitro* enzymatic assays (inhibitory activity against acetylcholinesterase (AChE), *α*-glycosidase, and *α*-amylase).

*Epipactis helleborine* and *N. ovata*: The rhizomes of these species were cited as medicinal, but information on compounds and biological properties is available only for the leaves. Mannose-specific lectins obtained from homogenate plant material were tested on MT-4, HEL, HeLa, and MDCK cell lines and showed several antiviral activities against HIV-1, HIV-2, CMV, RSV, and influenza A [170,171,172].

*Orchis mascula*: Ethanolic flower extracts showed saponins, flavonoids, anthraquinone, terpenoids, tannins, cyanogenic glycosides, and cardiac glycosides [226]. Compounds such as 2-methyl-Z,Z-3,13-octadecadienol, n-hexadecanoic acid, 2 furancarboxaldehyde 5-(hydroxymethyl), 2-propanone, 1,1-diethoxy, D-allose, 1,6-anhydro-*α*-D-galactofuranose, 3-acetylthymine, DL-4-amino-3-hydroxybutyric acid have been found. Antimicrobial activity against various pathogens such as *Salmonella paratyphi, Klebsiella oxytoca* or *Staphylococcus aureus* has been assessed.

## 4. Conservation Concerns and Action

According to Wraith and Pickering [7], biological use, which includes illegal collection of plants from the wild for medicine, food, and trade [265], is the most common threat for orchids globally, and particularly for terrestrial species. This was observed also for European species, since 12 out of the 16 species examined with threats were affected by biological use. Tuber collection, indeed, is still practiced, but for ornamental and private gardening uses in Italy and other developed European countries, with collectors often unaware of the identity and protection levels for orchid species ([24,25,26,27]; personal observations by M.B. and J.C). As mentioned above, in Europe, thanks to the Habitat Directive and the Natura2000 network [266], but also to regional and national level laws, the collection of threatened species is regulated or forbidden, and it is often mandatory to ask special permission from the local authorities for collecting plants or any portion of them.

As recently reviewed by Masters et al. [267], there are many Salep patents including both industrial and medicinal products, which are easily found even with a quick search on e-commerce platforms like eBay. As highlighted in Table 1, this traditional preparation is still very popular in Turkey, Greece, the Balkans, and in neighbouring Iran as well, increasing the risk of extinction for several species [24,25,26,27,139,165]. Indeed, it has been reported that thousands of individuals are harvested every year [81,165,268]. The practice is now forbidden in many Mediterranean countries and the product is proposed mainly with guar-gum (*Cyamopsis tetragonoloba* (L.) Taub.) as a substitute for tubers (or only as a starch pudding with vanilla flavour); other alternatives consist in Salep made with cereal starch or synthetic carboxymethyl cellulose [38,165]. However, illegal harvesting still occurs in Turkey, Greece, and Iran [13,25,163,165]. Ghorbani et al. [163,164] listed non-disruptive collection as a compromise to meet Salep request, with the possibility of introducing local bans in the case of substantial population decline. However, levels of sustainable harvesting are still under scientific debate, especially because there is, in general, an increasing demand of Salep made by the rising middle-class among developing countries, with a major request of exportation to Western nations (with Germany leading) that has been linked to illegal collection [27,81,163,164,269]. Kreziou et al. [24] noticed that there had been a change in the utilization of *Dactylorhiza* species from previous reports, and they hypothesized that this was caused by the increased rarity of the more desired *Anacamptis* and *Ophrys* following overharvesting.

It has been reported that in the late 1800s, harvesting was performed by selecting tubers by size, with small tubers being replanted to promote regrowth; in support to this, Sumpter et al. [270] provided experimental evidence of the regenerative capacity of old orchid tubers, while Caliskan et al. [131] found that *O. sphegodes* harvested at early flowering stage was able to develop new tubers. However, Kreziou et al. [24] recorded neither dispersal of young tubers nor replanting of old tubers in their study in North Greece.

*In vitro* propagation has been proposed in extra-EU countries as potential alternative to overcollection [271,272,273,274,275,276]. However, orchids used for Salep are generally difficult to propagate and cultivate on a commercial scale for their tubers. Currently, only a few small-scale cultivation trials for orchid tubers exist, therefore they are only available commercially via wild harvesting [24,165].

A few companies have started to propagate European orchids for ornamental purposes such as Albiflora (https://albiflora.be/index.php, accessed by 14 December 2022, Phytesia (http://www.phytesia.com/en/, accessed by 14 December 2022), or Bewdley Orchids (https://www.bewdleyorchids.com/, accessed by 14 December 2022). However, apart for the latter which specifies that local native orchids are propagated with the aim to help dwindling populations, other companies do not indicate the provenience of seed material. This sets potential problems of genetic pollution, increasing hybridization with local orchid species ([277] and references therein), and also the introduction of allochthonous microorganisms used in the potting mix, in particular bacteria and fungi, which could compete with the endophytes found in native populations, i.e., [278,279,280]. Given that recent studies seem to suggest that orchids can act as reservoir of orchid mycorrhizal fungi [281], conservation efforts would not only be pivotal for orchid populations persistence but also for the hosted microorganisms, which deserve more scientific attention as potential sources of active signalling molecules of ecological, agronomical, and industrial interest [263].

With the aim of helping local harvesters and resource managers to apply the best choices in terms of sustainability, very recently Ticktin et al. [282] reviewed existing data on the harvest pressure on orchids and consequent effects on their ecology and demography. These authors finally constructed a dichotomous key based on 12 characteristics, which can be used to determine in a local context whether and how a population could be subjected to a sustainable harvest. Characteristics include natural distribution and local abundance, habitat management, the existence of methods for the species cultivation, harvest type, and demand; for the development of the key, authors also considered characteristics of five Salep species (*A. morio, A. pyramidalis*, *D. sambucina, Orchis italica, O. mascula).*

## 5. Concluding Remarks

In conclusion, in this review we grouped citations of 62 orchids in the European folk tradition, and we examined the available information on phytochemicals and pharmacological activities for 85 species. As expected, the harvesting of tubers for Salep was highly mentioned and is still currently practiced: we confirm that there is no scientific evidence including pharmacological trials on humans that can justify the claimed aphrodisiac and healthy effects. Orchid tubers were found to contain interesting bioactive compounds that could at least partially explain the home consumption for medicinal purposes; however, also in this case, pharmacological confirmations are still lacking. Furthermore, it should be emphasised that tubers are heavily processed upstream of the final Salep powder, and that in chemical characterizations only compounds such as glucomannan, starch and ash were recognized. Claimed therapeutic effects of Salep should therefore be rather linked to mind suggestion and to the symbolic meaning that has been attributed to the testicle-like appearance of tubers for centuries. Currently, no efficient use of plant propagation methods has been found to fully supply Salep request: therefore, further studies are needed to better evaluate costs, benefits, and risks of an alternative production.

It should be mentioned that, as reported by Kreziou et al. [24], many Salep consumers were not aware that the product included material from threatened species. Conservation measures including dissemination/education programs aimed at raising public awareness on issues of nature protection are important to be considered and have already proved to be strategically useful (i.e., see [128,282]; www.globalorchidtrade.wixsite.com, accessed by 14 December 2022; www.lifeorchids.eu, accessed by 14 December 2022).

Compounds with demonstrated high therapeutic values have also been found in other orchid tissues less mentioned in the folk tradition: although even in these cases pharmacological trials need to be performed, leaves and flowers showed bioactive phytochemicals such as quercetin, kaempferol, chlorogenic acid, coumarins, phenanthrenes, alkaloids, and anthocyanins. Considering this, the possibility of deepening the study on orchid phytochemistry without exerting excessive pressure on individuals by harvesting, and possibly by obtaining material from *in vitro* micropropagation, may suggest ideas for new research and applications.

## Figures and Tables

**Table 1:** Traditional uses and conservation status of orchids distributed in Europe and Mediterranean.

Species	Portion/Preparation ^a^	Uses	Status (2021–2022) ^b^	Threatening Factors
*Anacamptis coriophora s.l*. (L.) R.M. Bateman, Pridgeon and M.W. Chase	S; T	Salep obtained from tubers used as medicinal food against cold, inflammation, cough and gastro-intestinal problems or as tonic, medicine, mind-developer, aphrodisiac (Bulgaria [26], Serbia [32], Turkey [33,34,35], and Greece [36]); in Turkey, tubers are used in decoction for strengthening and against wounds, abscess, inflammation, digestive diseases, and to improve circulatory system [34] and mental wellness [37]; Salep is used as food in Turkey [38,39].	LC (Europe). National protection in Belgium, France, Luxembourg. Regionally EX in Czech Republic, Estonia, Netherlands; CR in Germany; EN in Belarus, Switzerland; VU in Croatia and France; NT in Hungary. Regional protection in Italy [40].	Habitat conversion; extensive drainage, agricultural use of the habitat including fertilization and plant collection.
*Anacamptis* *laxiflora* (Lam.) R.M. Bateman, Pridgeon and M.W. Chase	S; T	Tuber used as medicinal food (Serbia [41,42], Turkey [43]); tuber’s mucilages used as astringent, expectorant, for anti-diarrhetic properties and for the treatment of bronchitis and convalescence (Southern Europe [44]). Tuber is used to produce Salep in Serbia [32] and Bulgaria [26].	LC (Europe), LC (Mediterranean). Protected species in Serbia; VU in the Bulgarian Checklist; protected in some regions of France (Champagne-Ardennes, Bourgogne, Franche-Comté, Rhone-Alpes, Provence-Alpes-Côte-d’Azur, Gironde). Regional protection in Italy [45].	Wetland decline, drainage, infilling, water abstraction for agricultural use, habitat urbanization, collection.
*Anacamptis morio s.l.* (L.) R.M. Bateman, Pridgeon and M.W. Chase,	S; T; FS; UN	Tubers in infusion as anti-hypertensive, against influenza, stomach disorders, for wound healing (Albania, South Kosovo and Balkans [46,47], Turkey [48]). Tuber used to treat cough, to strengthen sex potency, as tonic (Bosnia Herzegovina [49]). For the first date, boys offered girls tubers of this species in exchange of palmate orchid tubers (Northern Italy [50]). Tuber as food (Northern Italy [51,52]). Source of Salep in Bulgaria, Turkey [38,39,48]), Greece [27,36], and Serbia [32,41]. In Sardinia (Italy), flowering stems including those of subsp. *longicornu* were employed as ornamental in houses [53]. Tuber used for Salep in South Kosovo [54]. The species was cited among medicinal plants of Hungary [55] as well as those of Serbia [42] and the Carpathian area in the herbal of Stefan Falimirz (~1550 A.C.) (Central Europe [56]).	NT (Europe). Protected in Belgium, Luxembourg, France (some regions), Czech Republic, Slovakia. Included in national red lists. EN in Belarus, Czech Republic, Germany, Luxembourg; NT in Croatia, Norway, Switzerland, United Kingdom; LC in France. Regional protection in Italy [57].	Habitat conversion, degradation and decline, intensive use of fertilisers, drainage, deforestation, industrial development.
*Anacamptis* *palustris s.l.* (Jacq.) R.M. Bateman, Pridgeon and M.W. Chase	S; FS	Used in decoction for the treatment of respiratory disorders in Bulgaria, where the subspecies *elegans* is used to obtain Salep [26]. In Turkey the tuber is sold as source of Salep [35,38,39,58]. The flowering stem is cut and kept in vessels as ornamental in Turkey [59].	LC (Global), LC (Mediterranean). Protected in some regions of France. Regional protection in Italy [60].	Drainage, infilling, agriculture pressure on wetland habitats, intensive collection.
*Anacamptis* *papilionacea* (L.) *s.l.* R.M. Bateman, Pridgeon and M.W. Chase	S; FS	The tuber is used to produce Salep and against cough or as aphrodisiac (Turkey [25], Bulgaria [26], and Greece [36]). The flowering stem was collected and used as ornament in houses (Sardinia, Italy [53] and Turkey [25]); in Sicily (Italy), the flowering stem was employed in spells and evil eye [61].	LC (Europe). Protected in Belgium, Luxembourg, in some regions of France. VU in Bulgaria. Regional protection in Italy [62].	Animal grazing, agricultural and urbanisation pressures on habitat, fire, and deforestation.
*Anacamptis* *pyramidalis* (L.) Rich.	S; T; FS; W	Decoction of tubers used against cough, cold, flu, vasodilator, tonic, aphrodisiac (Bosnia-Herzegovina [49], Turkey [35,63,64,65], Bulgaria [26]). Tuber is used in infusion for pleasure and as psychedelic (Turkey [63]). Tuber as medicinal food (spice) or source of Salep (Serbia [41,42], Greece [24], and Turkey [38]. Whole plant is cultivated as ornamental in Turkey [66]; flowering stem is collected as decorative in Liguria (Italy (personal observation M.B., J.C.)).	LC (Europe). Protected in Belgium, Luxembourg, in some regions of France, Czech Republic, Slovakia. The Maltese variety *A. urvilleana* is listed in the Annex II of Habitat Directive; VU in Bulgaria. Regional protection in Italy [67].	Habitats (garrigue and steppe) in decline, often due to its classification as wasteland and to the spreading of invasive species (ruderals and aliens); urbanisation, quarrying, generic land use.
*Cypripedium* *calceolus* L.	R; RZ	Powdered roots and rhizome in sugar water were said to act as sedative, promote sleep, and reduce pain (Europe [11]). A tea prepared from roots is used to treat jangling nerves and headaches (European Russia [68]).	NT (Europe), LC (Global). Present in the Italian Red list (LC). [69,70]	Collection. Habitat decline due to inadequate forest management and to the use of pesticides/herbicides. Overgrazing or grazing abandonment, which can give rise to competition with other plant species, are both threating factors.
*Dactylorhiza* *baumanniana* J. Hölz. and Künkele	S	Tuber as source of Salep (Bulgaria [26]) and used to treat coughs, as aphrodisiac/tonic.	NT (Global and Europe) [71]	Habitat changes: soil drainage, tourism, reservoir construction, water insufficiency, pollution, overgrazing. High probability of hybridization.
*Dactylorhiza* *cordigera* (Fr.) Soó	S	Tuber as source of Salep (Bulgaria [26]): treatment of coughs, as aphrodisiac and tonic.	LC (Global and Europe) [72]	Habitat (wetlands) in decline; agricultural use of swampy fields, urbanisation, trampling; overgrazing; collection.
*Dactylorhiza euxina* (Nevski) Czerep	T; L	Tuber used in infusion in Turkey [73]: used to cure cough, inflammation and skin affections including boils; used as aphrodisiac, tonic. Leaves crushed and used to cure wounds (Turkey [74]).	NT (Global) [75]	Biological resource use.
*Dactylorhiza iberica* (M.Bieb. ex Willd.) Soó	S; T	Tuber as source of Salep, which is used as medicinal food and to treat cold, flu, rheumatism, and as a body warmer in Turkey [35,58,64,65,76].	VU (Europe) Listed as Endangered in the national Red List of Cyprus [77]	Ecological range is narrow and exposed to changes in hydrology by groundwater extraction. Other threats: competition with other species, hybridization and recreational activities, tourism development, urbanization, trampling, and plant collection.
*Dactylorhiza* *incarnata s.l.* (L.) Soó	S; T	Tuber as food (Northern Italy [52]); tuber used to produce Salep and taken as tonic, aphrodisiac (Bulgaria [26]).	LC (Europe). National protection in Belgium and Luxembourg, regional protection in France; CR in Bulgaria Luxembourg; EN in Croatia, Germany; VU in France; NT in Hungary, Switzerland; LC in Norway, United Kingdom. Regional protection in Italy [78]	Drainage, overgrazing, agriculture and urbanisation pressure, tourism, intensive recreation; high probability of hybridization.
*Dactylorhiza* *insularis* (Sommier) Landwehr	FS	Employed as decoration in houses in Nuoro province, Sardinia (Italy [53]).	Not Evaluated. National protection in Italy; total protection in Emilia Romagna (Italy); NT according to France’s Red List	
*Dactylorhiza* *kalopissii* E.Nelson	S	Tuber as source of Salep (Bulgaria [26]): coughs, aphrodisiac, tonic.	EN (Global, Europe and Mediterranean). CR in Bulgaria; present on Annex II of Habitat Directive [79]	Habitat (wetlands) in decline due to soil drainage, tourism, overgrazing, and reservoir construction; collection; high tendency to hybridization.
*Dactylorhiza* *maculata s.l.* (L.) Soó	S; T; FS	Tuber used for Salep in UK, as an aphrodisiac [14]; inflorescence was used as psychoactive (Valtellina, Northern Italy [80]). Tuber as food (Piedmont, Northern Italy [52]). Tuber recognized by DNA barcoding as component of Salep sold in Greece [81]. In Sardinia (Italy), this species was employed in rituals to reunite or separate couples [82].	LC (Europe). Protected in Belgium and Luxembourg. Regional protection in Italy [83].	Anthropogenic pressure on the habitats; drainage, urbanisation, tourism, trampling; collection.
*Dactylorhiza majalis* (Rchb.) P.F.Hunt and Sommerh	S; T	Tuber is used for Salep production in Turkey [84]. This species was cited as medicinal by Turner in 1568, while Langham in 1579 reported as antipyretic, anti-consumption and for anti-diarrhoeal effects [85].	LC (Europe). Protected in Belgium, Luxembourg, Czech Republic, Slovakia and in some regions of France and Italy [86].	Habitat menaced by anthropogenic threats including drainage, wrong management of wetlands, agricultural use of land, urbanization, tourism, trampling, plant collection.
*Dactylorhiza* *osmanica* (Klinge) P.F.Hunt and Summerh	S; T	Tuber as source of Salep [38], or as medicinal food used to cure coughs, inflammation, ulcers in Turkey [87] and skin affections including boils; used as aphrodisiac, tonic (Turkey [35,63,64,65,73]).	AS: Not Evaluated.	
*Dactylorhiza romana s.l.* (Sebast.) Soó	S	Tuber as source of Salep (Bulgaria [26], Turkey [38,88]): used as food and to cure coughs, or as aphrodisiac, tonic.	LC (Europe). VU in Bulgaria; present in the Italian Red List [89].	Trampling, agriculture, urbanisation, tourism; collection for Salep production.
*Dactylorhiza* *saccifera* (Brongn.) Soó	S; T	Tuber used in decoction as panacea, and breath shortness (Turkey [90]); tubers harvested for Salep (Greece [24,27]).	LC (Europe) [91].	Plant collection, habitats menaced by drainage, agricultural use, urbanization, tourism, deforestation
*Dactylorhiza* *sambucina* (L.) Soó	S; T; L; F	Leaves and flowers used in decoction for treatment of cough (Italy, Liguria, Val di Praglia [92]). Tuber used as food (Northern Italy [52]). Tuber prepared in Salep in Bulgaria [26] and in Greece [24,27]; tuber used to cure respiratory diseases (Macedonia, Greece [93]).	LC (Global and Europe). Regional protection in Italy [94].	Drainage, agriculture, urbanisation, tourism, trampling, deforestation, collection.
*Dactylorhiza* *umbrosa s.l.* (Kar. and Kir.) Nevski	S; T	In Turkey, tuber is prepared in infusion as tonic, or used to cure wounds, abscess, inflammation [73], or is a source of Salep [76].	Not evaluated.	
*Dactylorhiza* *urvilleana* (Steud.) H.Baumann and Künkele	T	In Turkey, tuber is prepared in infusion to treat skin problems such as wounds, abscess, inflammation, and as tonic [73,95].	LC (global) [96].	Over-grazing in some regions of the Caucasus.
*Epipactis helleborine s.l.* (L.) Crantz	RZ; R; L; UN	This species was cited by Rumphius in his *Herbarium Amboinense*, where he reported that the roots and rhizome were used in Europe to treat insanity and against rheumatisms [11]. The species is cited for the treatment of gout in European folklore [13]. In Sardinia, leaves were used to cure wounds [97]. Used as aphrodisiac (Mediterranean Europe [85]).	LC (Europe). LC in Czech Republic, Denmark, France, Germany, Hungary, Luxembourg, Norway, Switzerland, United Kingdom; EX in Cyprus. Regional protection in Italy [98].	Habitat in decline due to the coniferization or clearance of woodlands, use of soil, overgrazing; urbanisation, tourism, recreational activities.
*Gymnadenia* *conopsea* (L.) R.Br.	S; T; FS; UN	Decoction of tuber for treatment of lung diseases and strengthening of sexual potency (Bosnia-Herzegovina [49]). Tuber used as source of Salep or as medicine (Serbia [41,42]); flowering stem was collected for its perfume and used as ornamental [99]. In Abruzzo (Italy), tubers were employed in rituals to reunite or separate couples [82]. The species was cited among medicinal plants of the Carpathian area in the herbal of Stefan Falimirz (~1550 A.C) (Central Europe [56]).	LC (Europe). Regional protection in Italy [100].	Habitat conversion and decline due to ploughing, agriculture, abandonment of grazing, overgrazing, urbanisation, tourism.
*Gymnadenia rhellicani* (Teppner and E.Klein) Teppner and E.Klein	T; F; W	Flower distillate used as digestive (Northern Italy [51]). Oleolite of flowers was supposed to have aphrodisiac properties (Valtournanche, Italy [101]). Flowers are used to product cheese (Aosta Valley) and as flavouring in sweets (Lombardy) [102]; flowers are used against fever and respiratory diseases; liquoristic use is reported; aphrodisiac and ritual use (Aosta Valley, Italy [103,104]. In Piedmont, (North-Western Italy), as ritual, decoction of tubers was drunk in secret to reunite or separate couples, while flowers were used as ornament for S. Martino in Moena and Predappio (North-Eastern Italy) [82].	LC (Europe). Total protection in some regions of Italy; listed as Least Concern in the red lists of France, Germany, and Switzerland [105].	Habitat in decline due to ploughing, agriculture, conversion of meadows into arable land, grazing; urbanisation, infrastructures, tourism; plant collection.
*Himantoglossum* *affine* (Boiss.) Schltr.	S	Tuber used to produce Salep in Turkey [38,84].	EN (Europe) [106].	Plant collection for Salep production or for horticultural purposes; wrong forestry management; herbivory; tourism.
*Himantoglossum* *hircinum* (L.) Spreng.	S; T	Tubers used as source of Salep in Turkey [84]. This species was cited as medicinal by Turner in 1568, while Langham in 1579 reported as antipyretic, anti-consumption and for anti-diarrhoeal effects [85].	LC (Europe). EN in Luxembourg; VU in Germany and Switzerland; NT in the UK; LC in France [107].	Infrastructure expansion, urbanisation, and tourism; climate change with this species being sensitive to drought; plant collection; abandonment of grazing; competition with other species.
*Himantoglossum jankae* Somlyay, Kreutz and Óvári	S	Tuber as source of Salep (Bulgaria [26]): for coughs, as aphrodisiac, tonic.	Not evaluated.	
*Himantoglossum* *robertianum* (Loisel.) P. Delforge	S; T; FS; W	Tuber in decoction to prepare “Kasùgottu” in Sardinia as food supplement and tonic (Italy [108]; tuber used to produce Salep or in infusion as medicinal tea (Turkey [66]); roasted tuber as food alternative to potatoes in Sicily (Italy [109,110]); tuber used as tonic, aphrodisiac, for coughs and gastritis in Spain [111]. Tuber was prepared in infusion with milk or plain in Turkey [112]; flowering stem is used as decorative [25]; whole plant is collected as ornamental in Italy (personal observations M.B., J.C.).	LC (Europe), LC (Mediterranean). Regional protection in Italy; LC in France [113,114].	Infrastructure expansion, agriculture, climate change (extreme temperatures); urbanisation and tourism; plant collection.
*Neotinea* *tridentata* (Scop.) R.M. Bateman, Pridgeon and M.W. Chase	S; T	Tuber as food (Northern Italy [52]); tuber as source of Salep (Serbia [32], Bulgaria [26], and Turkey [33,38]).	LC (Europe). CR in Czech Republic; VU in Croatia, Germany, Switzerland; NT in France and Hungary; regional protection in Italy [115].	Collection of tubers or whole plant; digging and herbivory; grazing abandonment and use of fertilisers leading to changes in vegetation; habitat in decline due to agricultural uses of land and ploughing; urbanisation, infrastructures, tourism.
*Neotinea* *ustulata* (L.) R.M. Bateman, Pridgeon and M.W. Chase	S; T	Tuber as food, tonic, aphrodisiac in Bulgaria [26]; tuber used to produce “Kasùgottu” in Sardinia (Italy [108]); tuber as source of Salep in Serbia [32].	LC (Europe). EX in Luxembourg; CR in Belarus; EN in Germany and the United Kingdom; VU in Bulgaria and Denmark; NT in Hungary; LC in France; regional protection in Italy [116].	Agriculture, inadequate ploughing, changes in habitat management, use of fertilisers, which favour the spreading of competing plants, climate change (extreme temperatures).
*Neottia ovata* (L.) Bluff and Fingerh	S; RZ; UN	A tincture of rhizome was used for improving skin tone and for the treatment of gastrointestinal problems (Spain [117]); the species was cited in *Gerard’s Herbal* in 1597 for the cure of green wounds, bursting and ruptures [13] and in the Sussex list of wound cures (Great Britain [118]; rhizome was used to produce Salep in the Highlands (Great Britain [118]).	LC (Europe). Listed as Near Threatened in the national Red List of Belarus [119].	Extreme temperatures; agricultural use of lowland, changes in forest management and deforestation; urbanisation, trampling, tourism.
*Ophrys* *apifera* Huds.	S; T; FS	Tuber in decoction (Kasùgottu) as food supplement and tonic in Sardinia, Italy [82,108]; in this region it was also cited as anti-inflammatory gastrointestinal, against childhood diarrhoea, cystitis, and nephritis [120]. The tuber is used for Salep production in Turkey [84]. This species was cited as medicinal by Turner in 1568, while Langham in 1579 reported as antipyretic, anti-consumption and for anti-diarrhoeal effects [85]; flowering stem is harvested as ornamental in Genoa (Italy (personal observation M.B. and L.C.)).	LC (Europe). Total protection in some regions of Italy [121].	Urbanisation, infrastructures; plant collection and herbivory.
*Ophrys* *argolica s.l.* Griechenland	FS	Flowering stem from subsp. *lesbis* used as decorative in Turkey [25].	VU (global, Europe, Mediterranean). Protected at national level (67/81) and listed as VU in the Greek Red List [122].	Urbanisation, construction work, residential building, and tourism. The use of herbicides and pesticides negatively affects the pollinators of this species.
*Ophrys fusca s.l.* Link.	S	Used for Salep (Turkey [38,76]).	LC (Europe). VU on the Croatian Red List; Total protection in some regions of Italy [123].	Urbanisation, buildings; plant collection.
*Ophrys holosericea s.l.* (Burm.f.) Greuter	S; T	This species was cited as medicinal by Turner in 1568, while Langham in 1579 reported as antipyretic, anti-consumption and for anti-diarrhoeal effects [85]; tuber used for Salep production (Turkey [38]).	LC (Europe). Total protection in some regions of Italy; VU on the Croatian Red List [124].	Urbanisation, construction work, residential building, and plant collection.
*Ophrys* *insectifera* L.	T	This species was cited as medicinal (syn. *O. muscifera)* by Turner in 1568, while Langham in 1579 reported as antipyretic, anti-consumption and for anti-diarrhoeal effects [85].	LC (Europe). Listed as CR on the Bulgarian red list; VU on the Croatian Red list; LC on the French Red List [125].	Urbanisation, buildings.
*Ophrys* *lutea s.l.* Cav.	T	Tuber prepared in infusion with milk or plain in Turkey [112].	LC (Europe). Listed as EN in Croatia and as LC on the French Red List; total protection in some regions of Italy [126].	Urbanisation, residential building; plant collection.
*Ophrys* *reinholdii* H. Fleischm	S	Used to prepare Salep against cold, flu, and as body warmers, psychedelics, pleasure and medicinal tea (Turkey [63]); flowering stem used as decorative (Turkey [25]).	LC (Europe). [127].	Small size of the local populations; habitats menaced by urbanisation, construction work and residential building; tourism, plant collection; intensive traffic.
*Ophrys scolopax s.l.* Cav.	S	Used to prepare Salep against cold, flu, and as body warmers, psychedelics, pleasure and medicinal tea (Turkey [63]); in Villarino de los Aires (Spain) the whole plant is collected for its shape, or as a souvenir, or as a talisman, while in previous years it was hunted and given by boys to the girls they were interested in [128].	LC (Europe). LC in France; total protection in some regions of Italy [129].	Urbanisation, infrastructures, plant collection, and herbivory.
*Ophrys speculum* Link.	W	Whole plant was harvested for ornamental purposes in Genoa (Italy [99]).	LC (Europe). Total protection in some regions of Italy; VU in France’s Red List [130].	Urbanisation, construction work, residential buildings; plant collection.
*Ophrys sphegodes s.l.* Mill.	S; T; FS	Used for Salep (Turkey [38,131]). This species was cited as medicinal by Turner in 1568, while Langham in 1579 reported it as antipyretic, anti-consumption and for anti-diarrhoeal effects [85]. Flowering stem of *O. sphegodes* subsp. *atrata* was used as decoration in Sardinia (Italy [53]).	LC (Europe). Total protection in some regions of Italy [132].	Urbanisation, infrastructures, plant collection and herbivory.
*Ophrys* *tenthredinifera* Willd	FS	Tubers harvested for Salep; flowering stem used as decorative (Turkey [25]).	LC (Europe). Total protection in some regions of Italy [133].	Habitat loss due to urbanisation, construction work, residential building; plant collection.
*Ophrys* *umbilicata* Desf.	S	Used for Salep (syn. *Ophrys attica*) and consumed as hot drink with milk in Western and Central Anatolia (Turkey [76]).	LC (Europe) [134].	Plant collection and habitat disturbance by anthropogenic activity such as urbanisation, buildings, tourism.
*Orchis* *anatolica* Boiss.	S; T	Used for Salep and consumed as hot drink with milk in Western and Central Anatolia (Turkey [38,76]); Salep used to treat cold, flu, as body warmers, chest pain, as psychedelics, for pleasure and medicinal tea in Turkey [63,135,136]. In Turkey, used to treat diarrhoea [137] and in decoction to cure stomach-ache and cough, as expectorant, against chest pain, as emollient, tonic, lactagogue [135,136].	LC (Global and Europe) [138].	Plant collection; risk of hybridization with *Orchis quadripunctata* on Cyprus and Crete. Tourism, land use changes, deforestation, expansion of villages and infrastructures.
*Orchis* *anthropophora* (L.) All.	S; T	Tuber as source of Salep (Turkey [139] and Greece [36]); tuber used as medicinal food for the treatment of respiratory diseases (Sardinia, Italy [97,140]).	LC (Europe). National protection in Belgium, Luxembourg, United Kingdom; regional protection in France and Italy [141].	Competition with other plants and shrubs; overgrazing; ploughing; infrastructures; tourism; inadequate timing of road verges clearing, use of pesticides and herbicides; herbivory.
*Orchis* *italica* Poir.	S; FS	Tuber is used to produce Salep (Turkey [38] and Greece [36]), which is employed to treat cold, flu, as body warmers, psychedelics, pleasure and medicinal tea [63,65]; flowering stem used as decorative [25].	LC (Europe) [142].	Urbanisation, buildings, expansion of infrastructure, overgrazing, deforestation, tourism, plant collection.
*Orchis mascula s.l.* (L.) L.	S; T; R; L; FS; UN	Tuber as food (Turkey [63,64]). Tuber as demulcent, astringent, tonic and nutrient, used to treat diarrhoea (Turkey [143]). Roots used as demulcent (Great Britain [13]); dried roots roasted and eaten (Peloponnesus, Greece [13,144]). Tuber used for Salep in Greece [24,27,36], Turkey [38,39], and Serbia [32]. Leaves are cooked with onion and eggs in Turkey [66]. This species was cited as aphrodisiac in Sardinia [97], as medicinal by Guarrera [82], and Turner in 1568, while Langham in 1579 reported as antipyretic, anti-consumption and for anti-diarrhoeal effects [85]. The flowering stem was employed in rituals in Italy (Vara Valley, Liguria [145]) for the protection of children against snakes and other animals. The species was cited among medicinal plants of the Carpathian area in the herbal of Stefan Falimirz (~1550 A.C.) (Central Europe [56]).	LC (Europe). National protection in Belgium, Luxembourg; regional protection in France and Italy [146].	Wrong woodland management (coniferization included); ploughing and agricultural uses of grasslands; overgrazing; fertilisers; plant collection; infrastructures; fires; tourism.
*Orchis* *militaris* L.	S; T	Tuber used for Salep in Greece [24]. Tuber as food (Northern Italy [52]). Tuber as medicinal (Serbia [41,42]).	LC (Europe). EN in Bulgaria; National protection in Belgium, Luxembourg, United Kingdom; regional protection in France and Italy [147].	Pressure on species’ habitat: use of fertilisers, overgrazing, drainage, ploughing, infrastructures, tourism, herbivory. Habitat conversion due to the abandonment of rural activities, which causes the rising of competing species and shrub development.
*Orchis* *pallens* L.	S	Tuber as source of Salep and used to treat cough or as aphrodisiac (Bulgaria [26] and Serbia [32]).	LC (Europe). Regional protection in Italy [148].	Changes in habitat due to the abandonment of the practice of coppicing and to the decrease in the amount of light available. Drought, late frost, animals digging and herbivory; urbanisation and infrastructures; tourism, deforestation, plant collection.
*Orchis* *provincialis* Balb. ex Lam. and DC.	S	Salep obtained from tubers used to treat cough and as aphrodisiac (Bulgaria [26], Greece [24]).	LC (Europe). CR in Bulgaria; listed in the Appendix I of the Berna Convention [149].	Changes in woodland composition leading to decreases in the amount of light; urbanisation; infrastructures; tourism; plant collection.
*Orchis punctulata* Steven ex Lindl.	S; T	Salep obtained from powdered tubers is mixed with milk and used as food in Turkey [35]. Infusion of tuber is employed to cure cold, flu, and as body warmers, psychedelics, pleasure and medicinal tea in Turkey [63].	VU (Europe). Included in the Red Data Book of Ukraine and Greece [150].	This species is very rare, and its habitat is menaced by several anthropogenic threats like fires, grazing, deforestation, tourism, urbanisation, buildings/infrastructure, and plant collection especially in unprotected sites.
*Orchis* *purpurea* Huds.	S; T	Tuber as medicinal food (Italy [52]). Tuber used for Salep (Serbia [32], Turkey [76]). In Abruzzo (Italy), tubers from this species were employed in rituals to reunite or separate couples [151].	LC (Europe). National protection in Belgium, Luxembourg; regional protection in Italy and France [152].	Harvesting of tuber for Salep production, animals digging, herbivory; ploughing, agriculture, use of fertilisers; urbanisation, tourism, infrastructures, deforestation.
*Orchis simia* Lam.	S; T	Tuber as source of Salep (Serbia [32], Turkey [38]), and Greece [24]), which is also used to treat cough or as tonic/aphrodisiac (Mediterranean area, Bosnia-Herzegovina [49]). Tuber as food (Northern Italy [52]). Tuber as medicinal in Serbia [41,42]. Tubers used in infusion to treat cold, flu, and as body warmers, psychedelics, pleasure and medicinal tea in Turkey [63]. This species was cited as medicinal by Turner in 1568, while Langham in 1579 reported as antipyretic, anti-consumption and for anti-diarrhoeal effects [85].	LC (Europe). National protection in Belgium, Luxembourg; regional protection in Italy and France [153].	Harvesting of tuber for Salep production; horticulture; animal digging, herbivory; overgrazing; habitat in decline for changes in woodland management, reforestation, ploughing, agriculture, use of fertilisers; urbanisation, tourism, infrastructures.
*Orchis* *spitzelii* Saut. ex W.D.J. Koch	S; T	Cough, tonic, aphrodisiac (Bulgaria [26]); tuber as source of Salep (Turkey [38]); tuber prepared in infusion with milk or plain in Turkey [112].	NT (Europe). EX in Germany; CR in Bulgaria, Spain, Switzerland; EN in Croatia; VU in Sweden; LC in France; regional protection in Italy [154].	Damage to populations due to annual winter sport activities and tourism; collection for decorative purpose and for Salep production; animal digging and herbivory.
*Platanthera bifolia* (L.) Rich	S; T; L	Tuber used to treat cough (Bosnia and Herzegovina [49]), cold, flu, body warmers; tuber used for Salep (Turkey [13]); tuber as medicinal food in Serbia [41,42]. In North-Eastern Italy, leaves were used against rheumatism and as antineuralgic, by putting swell dry leaves in water–vinegar and by applying swollen leaves to the affected area [155].	LC (Europe). VU in Belarus, Czech Republic; Germany, Luxembourg; NT in Croatia, Hungary, United Kingdom; LC in Denmark, France, Norway, Switzerland [156].	Destruction of habitats; “coniferization” of woodland; urbanisation and infrastructure development; agricultural use of the habitat. Disturbance to pastures and hay meadows; lack of grazing or overgrazing on the other hand. Collection of the species for ornamental purposes
*Platanthera* *chlorantha* (Custer) Rchb.	T; AP	Tuber as medicinal food (Serbia [41,42]); in Dorset (Great Britain [118]) aerial parts were used to produce an ointment to be applied on ulcers.	LC (Europe) VU in Belarus, Czech Republic, Germany, Luxembourg; NT in Croatia, Hungary, and in United Kingdom; LC in Denmark, France, Norway, and Switzerland [157].	Clearance or “coniferization” of woodland; destruction of grassland by agricultural improvement, urban and infrastructures; a switch to a dense woodland canopy is detrimental for flowering as the species needs light. Lack of grazing; increased competition with other plant species; overgrazing.
*Serapias* *lingua* L.	S; T	Tuber is used to produce Salep (Turkey [84]). This species was cited as medicinal by Turner in 1568, while Langham in 1579 reported as antipyretic, anti-consumption and for anti-diarrhoeal effects [85].	LC (Europe). NT in the National Red list of France [158].	Plant collection for Salep production, wild animals digging, ploughing and agricultural use of land; urbanisation; tourism; infrastructure development.
*Serapias vomeracea* (Burm. f.) Briq.	S; T; FS	Tuber used for Salep which is consumed against cold, flu, and as body warmer (Turkey [33,38,39,139]). This species was cited as medicinal by Turner in 1568, while Langham in 1579 reported as antipyretic, anti-consumption and for anti-diarrhoeal effects [85]; flowering stem is collected as decorative in Liguria (Italy (personal observation M.B, J.C.))	LC (Mediterranean). Listed as EN in the national Red Lists of Bulgaria, Malta, and Switzerland. VU in Croatia, LC in France [159].	Agricultural practices (draining of plains and meadows); mild collection for horticultural and digging/consumption by animals. Overgrazing, urbanisation and infrastructure expansion; tourism.
*Traunsteinera* *globosa* (L.) Rchb.	T	Tuber as food (Northern Italy [52]).	LC (Europe) CR in Bulgaria; EN in Czech Republic and Hungary; LC in France, Germany, and Switzerland [160].	Overgrazing, ploughing of meadows, urbanisation; tourism.
*Spiranthes spiralis* (L.) Chevall.	RZ; FS; W	Hypogean portions used as strong aphrodisiac (Europe [11]); flowering stem was smell as a tonic; whole plant was harvested and used for ornamental purposes (Genoa, Italy [99]).	LC (Europe). EX in Denmark; CR in Czech Republic; VU in Bulgaria; NT in France, Hungary, Switzerland, and United Kingdom [161].	Menaced by competition with more vigorous grasses and herbs; changes in habitat management and use of fertilisers; ploughing, overgrazing, mowing of pastures, urbanisation, tourism; plant collection.

^a^ S: hypogean portion used for Salep; T: tuber; RZ: rhizome; R: roots; L: leaves; AP: aerial parts; FS: flowering stem; F; flowers; W: whole plant; UN: unspecified. ^b^ IUCN risk categories: LC = Least Concern; NT = Near Threatened; VU = Vulnerable; EN = Endangered; CR = Critically Endangered; EX = Extinct in the Wild.

**Table 2:** Phytochemistry and evaluation of biological activities of European terrestrial orchids.

Species	Compounds	Plant Portion	Sample Type	Characterization	Biological Activity	References
*Anacamptis coriophora s.l.* (L.) R.M. Bateman, Pridgeon and M.W. Chase	Chrysanthemin, ophrysanin. Both the anthocyanins were detected in different amounts depending on the origin of the sample (Greece, Italy, Austria).	Flowers	Methanol extract	TLC, HPLC, and thin-layer electrophoresis		[168]
	Methyl-(E)-*p*-methoxycinnamate, 13-heptadecyn-1-ol, 2,5-dimethoxybenzyl alcohol, 4-(1,1,3,3-tetramethylbutyl)-phenol; 7,9-di-tert-butyl-1-oxaspiro (4,5) deca-6,9-diene-2,8-dione; 10-dodecenol, *p*-cresol, methyl (Z) *p*-methoxycinnamate, 2-dodecenal, methyl cinnamate.	Inflorescence bearing mature seeds	EOs isolated by hydrodistillation from *A. coriophora* subsp. *fragrans*	GC-MS analysis	Antioxidant activity (DPPH assay); anti-proliferative effect on carcinoma cells BxPC3 and 2008 human ovarian cancer cells	[174]
	Saturated hydrocarbons (heneicosane, nonadecane, tricosane, pentacosane, heptacosane); unsaturated hydrocarbons (9-pentacosene 9-heptacosene; 9-tricosene; 1-hexadecene); aldehydes (nonanal, phenylacetaldehyde, anisaldehyde); alcohols (2,5-dimethoxybenzyl alcohol) and terpenes (thymol and *α*-copaene).	Inflorescence	EOs isolated by steam distillation from *A. coriophora* subsp. *fragrans*	GC-FID and GC-MS analyses	Antioxidant activity (DPPH assay)	[175]
*Anacamptis laxiflora* (Lam.) R.M. Bateman, Pridgeon and M.W. Chase	Orchicyanin I, ophrysanin, orchicyanin II, seranin, serapianin, chrysanthemin, cyanin, unknown compounds.	Flowers	Methanol extract	TLC, HPLC, and thin-layer electrophoresis		[168]
*Anacamptis* *morio s.l.* (L.) R.M. Bateman, Pridgeon and M.W. Chase,	Isoquercitrin, caffeic acid, *p*-coumaric acid, chlorogenic acid and three unindentified flavonoid eterosides (aqueous fraction); caffeic acid, chlorogenic acid, isoquercitrin (hydromethanolic fraction); sitosterol and traces of pentacyclic triterpenoids (lipophilic fraction).	Leaves	Hydroalcoholic extract and relative fractions	Preparative HPLC		[176]
	Two unidentified glucosidic flavonoids, coumarin derivatives, *p*-coumaric acid, chlorogenic acid (aqueous fraction); caffeic acid and *p*-coumaric acid (hydromethanolic fraction); sitosterol and traces of pentacyclic triterpenoids (lipophilic fraction).	Flowering stem	Hydroalcoholic extract and relative fractions	Preparative HPLC		[176]
	Quercetin	Leaves	Methanol extract	Two-dimensional-paper chromatography (2D-PC) and UV spectral analysis		[169]
	Orchicyanin II, orchicyanin I, cyanin (from *A. morio* and *A. morio* subsp. *longicornu*).	Flowers		Chemical hydrolysis, absorption spectroscopy, chromatography		[177]
	9,10-Dihydrophenanthrenes (orchinol and hircinol).	Tuber	Enzyme preparations from induced orchid bulbs to accumulate phytoalexins	2D-PC, thin-layer chromatography and paper chromatography (TLC)		[178]
	Orchicyanin I, orchicyanin II, ophrysanin and lower amounts of chrysanthemin, cyanin, seranin.	Flowers	Methanol extract	TLC, HPLC, and thin-layer electrophoresis		[168]
	Glucomannan, starch.	Tuber	Tuber boiled in milk, dry and powdered	Gravimetric methods, protein characterization with Kjeldahl method		[38]
*Anacamptis* *palustris s.l.* (Jacq.) R.M. Bateman, Pridgeon and M.W. Chase	9,10-Dihydrophenanthrenes (orchinol and hircinol).	Tuber	Enzyme preparations from induced orchid bulbs to accumulate phytoalexins	2D-PC and TLC		[178]
	Orchicyanin I, ophrysanin, seranin, cyanin, chrysanthemin, serapianin, orchicyanin II.	Flowers	Methanol extract	TLC, HPLC, and thin-layer electrophoresis		[168]
*Anacamptis papilionacea s.l.* (L.) R.M. Bateman, Pridgeon and M.W. Chase	Quercetin.	Leaves	Methanol extract	2D-PC and UV spectral analysis		[169]
	Loroglossin, flavonoid heterosides.	Leaves	Hydroalcoholic extract			[179]
	9,10-Dihydrophenanthrenes (orchinol and hircinol).	Tuber	Enzyme preparations from induced orchid bulbs to accumulate phytoalexins	2D-PC and TLC		[178]
	Chrysanthemin, cyanin, seranin, ophrysanin, orchicyanin II, serapianin, orchicyanin I.	Flowers	Methanol extract	TLC, HPLC, and thin-layer electrophoresis		[168]
*Anacamptis* *pyramidalis* (L.) Rich.	High content in flavonoids, carotenoids; chlorophyll a-b and reduced glutathione.	Tuber powdered	Ethanol extract	Phytochemical screening by *in vitro* assays	High scavenging and antioxidant activity tested on SOD, CAT, GPx, and GSH-Px	[180]
	Saturated hydrocarbons (tricosane, pentacosane, heneicosane, heptacosane); aldehydes (nonanal, heptanal, phenylacetaldeide, octadecanal); alcohols (2-phenylethanol, benzyl alcohol); unsaturated hydrocarbons (9-pentacosene, 9-heptacosene, 7-heptacosene); acids (heptanoic acid, nonanoic acid); terpenes (*α*-copaene, thymol, *α*-cadinene).	Inflorescence	EOs isolated by steam distillation	GC-FID and GC-MS analyses	Antioxidant activity (DPPH assay)	[175]
	Flavonoids; disaccharide, citric acid, roseoside, gastrodin derivativs, dihydrohybenzoic acids; caffeic acid, acacetin derivatives; oxo-dihydroxy-octadecenoic acid; trihydroxy-octadecenoic acid.	Tuber	Methanol extract	Phytochemical screening by *in vitro* methods and HPLC-MS	High antioxidant activity and inhibitory potential against tyrosinase, *α*-amilase, and *α*-glucosidase	[181]
	Flavonoids; gastrodin derivatives; dihydroxybenzoic acid derivative; caffeic acid derivatives; acacetin derivatives; parishin; citric acid; disaccharide.	Tuber	Water extract	HPLC-MS	Inhibitory activity against tyrosinase, *α*-amilase, and α-glucosidase	[182]
	Chrysanthemin, cyanin, seranin, ophrysanin, orchicyanin II, serapianin, orchicyanin I.	Flowers	Methanol extract	TLC, HPLC, and thin-layer electrophoresis		[168]
*Calypso bulbosa* (L.) Oakes	Chrysanthemin, cyanin.	Flowers		Chemical hydrolysis, absorption spectroscopy, chromatography		[177]
*Cephalanthera damasonium* (Mill.) Druce	Quercetin.	Leaves	Methanol extract	2D-PC and UV spectral analysis		[169]
*Cephalanthera longifolia* (L.) Fritsch	Quercetin, kaempferol-O-glycosides.	Leaves	Methanol extract	2D-PC and UV spectral analysis		[169]
	Alkaloids.					[182]
*Cephalanthera rubra* (L.) Rich.	Quercetin.	Leaves	Methanol extract	2D-PC and UV spectral analysis		[169]
*Cypripedium* *calceolus* L.	Chrysanthemin, cyanin, seranin, ophrysanin, orchicyanin II, serapianin, orchicyanin I.	Flowers	Methanol extract	TLC, HPLC and thin-layer electrophoresis		[168]
	Linalool; octyl acetate; 32 terpenoid derivatives; 22 aliphatic and 18 aromatic compounds.	Inflorescence	Floral scent	DHS-GC-MS analysis		[183]
	Chrysanthemin.	Flowers		Chemical hydrolysis, absorption spectroscopy, chromatography		[177]
*Dactylorhiza baumanniana* J. Hölzinger and Künkele	Cyanin, orchicyanin I, orchicyanin II, serapianin, seranin, ophrysanin, chrysanthemin.	Flowers	Methanol extract	TLC, HPLC, and thin-layer electrophoresis		[168]
*Dactylorhiza* *foliosa* (Rchb.f.) Soó	Orchicyanin I, orchicyanin II, cyanin, serapianin, seranin, chrysanthemin.	Flowers	Methanol extract	TLC, HPLC, and thin-layer electrophoresis		[168]
*Dactylorhiza iberica* (M.Bieb. ex Willd.) Soó	Orchicyanin I, orchicyanin II, serapianin, cyanin, seranin.	Flowers	Methanol extract	TLC, HPLC, and thin-layer electrophoresis		[168]
*Dactylorhiza* *incarnata s.l.* (L.) Soó	6-hydroxyflavones.	Leaves	Methanol extract	2D-PC and UV spectral analysis		[169]
	Orchicyanin I, serapianin, orchicyanin II, seranin, cyanin.	Flowers	Methanol extract	TLC, HPLC, and thin-layer electrophoresis		[168]
	4-hydroxyphenylacetic acid methyl ester, 4-hydroxybenzyl alcohol, 4-hydroxybenzaldehyde and two unidentified compounds as predominant.	Flowers	Trimethylsilylated dichloromethane extract	GC-MS analysis		[184]
	Acids (mainly palmitic acid, nonanoic acid, lactic acid, caproic acid); benzoids (mainly *p*-hydroxybenzyl alcohol, *p*-coumaric acid, hydroquinone, 3,4-dihydroxybenzyl alcohol).	Flowers; compounds from both var. *incarnata* and subsp. *ochroleuca*	Floral scent diethyl ether extract	GC-MS analysis		[185]
*Dactylorhiza* *kalopissi* E. Nelson	Orchicyanin I, orchicyanin II, cyanin, serapianin, seranin, ophrysanin.	Flowers	Methanol extract	TLC, HPLC, and thin-layer electrophoresis		[168]
*Dactylorhiza maculata s.l.* (L.) Soó	Quercetin, kaempferol.	Leaves	Methanol extract	2D-PC and UV spectral analysis		[169]
	Orchicyanin II, orchicyanin I, cyanin.	Flowers		Chemical hydrolysis, absorption spectroscopy, chromatography		[177]
	Orchicyanin I, orchicyanin II, cyanin, serapianin, seranin, chrysanthemin, ophrysanin.	Flowers	Methanol extract	TLC, HPLC, and thin-layer electrophoresis		[168]
	4-hydroxybenzyl alcohol, hydrocinnamic acid, 4-hydroxyphenylacetic acid methyl ester, nonanoic acid, nonanal, hexanoic acid; 4-hydroxybenzaldehyde and two unidentified compounds as predominant.	Flowers from subsp. *fuchsii*	Trimethylsilylated dichloromethane extract	GC-MS analysis		[184]
	4-hydroxyphenylethanol, 4-hydroxybenzyl alcohol, 4-hydroxyphenylacetic acid methyl ester, 4-hydroxybenzaldehyde, nonanoic acid, and three unidentified compounds as predominant.	Flowers from subsp. *maculata*	Trimethylsilylated dichloromethane extracts	GC-MS analysis		[184]
	*p*-hydroxybenzyl alcohol, 3,4-dihydroxybenzyl alcohol, hydroquinone, *p*-coumaric acid, palmitic acid.	Flowers from subsp. *fuchsii*	Floral scent extracted with diethyl ether	GC-MS analysis		[185]
	Rutin, quercetin, 3,3′,4′,5,5′,7-hexahydroxyflavonone, 3,3′,4′,5,5′,7-hexahydroxyflavonone-3-O-glycoside, gallic acid, and ferulic acid as predominant compounds.		Ethanol extract	Preparative HPLC and HPLC-DAD analysis	Antioxidant and antibacterial activity	[186]
*Dactylorhiza majalis s.l.* (Rchb.) P.F. Hunt & Summerh.	Nonanoic acid, two unidentified aromatic compounds, 4-hydroxybenzyl alcohol, 4-hydroxybenzaldehyde, 4-hydroxyphenylacetic acid methyl ester, 2-decenal, nonanal.	Flowers	Trimethylsilylated dichloromethane extracts	GC-MS analysis		[184]
	Orchicyanin II, orchicyanin I, cyanin.	Flowers		Chemical hydrolysis, absorption spectroscopy, chromatography		[177]
	Orchicyanin I, orchicyanin II, serapianin, seranin, cyanin, chrysanthemin, ophrysanin.	Flowers	Methanol extract	TLC, HPLC, and thin-layer electrophoresis		[168]
	Acids (mainly lactic acid, nonanoic acid, caproic acid, palmitic acid, phosphoric acid), benzoids (mainly *p*-hydroxybenzyl alcohol).	Flowers	Floral scent diethyl ether extract	GC-MS analysis		[185]
*Dactylorhiza* *osmanica* (Klinge) P.F. Hunt and Summerh	Orchicyanin I, orchicyanin II, cyanin, serapianin, ophrysanin.	Flowers	Methanol extract	TLC, HPLC, and thin-layer electrophoresis		[168]
	Protease enzyme.	Tuber (powdered)	Protein fraction	SDS-PAGE; gel filtration chromatography analysis	Hydrolisation of *α*-, *β*-, and *κ*-casein	[187]
	Flavonoids; fumaric acid, *p*-coumaric acid, quercitrin, and vanillic acid, ascorbic acid as the most abundant polyphenols.	Flowering stem	Ethanol extract	Phytochemical screening by *in vitro* assays and HPLC–HRMS analysis	Antioxidant activity; inhibitory activity against AChE, *α*-glycosidase, and *α*-amylase	[188]
	Flavonoids; fumaric acid, p-coumaric acid, rosmarinic acic and vanillic acid as the most abundant polyphenols.	Tuber	Ethanol extract	Phytochemical screening by *in vitro* assays; HPLC–HRMS analysis	Antioxidant activity, anti-Alzheimer and anti-diabetes properties evaluated by enzymatic assays (inhibitory activity against acetylcholinesterase (AChE), *α*-glycosidase, and α-amylase)	[188]
*Dactylorhiza praetermissa* (Druce) Soó	6-hydroxyflavones.	Leaves	Methanol extract	2D-PC and UV spectral analysis		[169]
*Dactylorhiza* *romana s.l.* (Sebast.) Soó	Cyanin, seranin, orchicyanin II, serapianin, orchicyanin I.	Flowers	Methanol extract	TLC, HPLC, and thin-layer electrophoresis		[168]
	*β* -pinene, sabinene, limonene, *β*-phellandrene, (E)-ocimene and trans-sabinene hydrate, linalool, benzaldehyde.	Inflorescence	Floral odours collected from three different morphs with Porapak Q filters	GC-MS analysis		[189]
	Benzoic acid and protocatequic acid, luteolin, *p*-hydroxy benzoic acid as the most abundant ones in the ethylacetate extract); flavonoids.	Tuber	Hexane, ethyl acetate, chloroform, ethanol and methanol extracts	Phytochemical screening by *in vitro* assays and HPLC-DAD analysis	Antioxidant activity (ABTS, DPPH, and FRAP assays); evaluation of *α*-amylase and *α*-glucosidase inhibition activity; antimicrobial activity	[190]
	*p*-hydroxybenzoic acid, kaempferol, rosmarinic acid, caffeic acid as the most abundant compounds.	Tuber	Ethanol extracts	Phytochemical screening by *in vitro* assays and HPLC-DAD analysis	Antioxidant activity (ABTS, DPPH, and FRAP assays); evaluation of *α*-amylase and *α*-glucosidase inhibition activity; antimicrobial activity	[191]
*Dactylorhiza* *saccifera* (Brongn.) Soó	Orchicyanin I, cyanin, orchicyanin II, serapianin, seranin.	Flowers	Methanol extract	TLC, HPLC, and thin-layer electrophoresis		[168]
	Caryophyllene, pentadecane, hexadecane, and heptadecane.	Inflorescence	Floral scent	HS-SPME-GC–MS analysis		[192]
*Dactylorhiza sambucina* (L.) Soó	Quercetin-3-glucoside; quercetin-7-glucoside; quercetin-3,7-diglucoside, caffeoil-1-glucose, chlorogenic acid, *p*-cumaroil-1-glucose; coumarin derivatives in traces.	Leaves	Hydroalcoholic extract and relative fractions	Preparative HPLC		[176]
	Isoquercitrin, quercetin 3,7-diglucoside, chlorogenic acid, *p*-coumaric acid and caffeic acid; 6-metossi-7-idrossi-coumarin.	Flowers	Hydroalcoholic extract and relative fractions	Preparative HPLC		[176]
	Orchicyanin II, orchicyanin I, cyanin.	Flowers		Chemical hydrolysis, absorption spectroscopy, chromatography		[177]
	Cyanin, seranin, ophrysanin, orchicyanin II, serapianin, orchicyanin I.	Flowers	Methanol extract	TLC, HPLC, and thin-layer electrophoresis		[168]
	Caryophyllene, *β*-sesquiphellandrene, 4,5-di-epi-aristolochene.	Inflorescence	Floral scent	HS-SPME-GC–MS analysis		[192]
*Dactylorhiza* *umbrosa* (Kar. and Kir.) Nevski	Orchicyanin I, orchicyanin II, serapianin, cyanin, seranin, chrysanthemin.	Flowers	Methanol extract	TLC, HPLC, and thin-layer electrophoresis		[168]
	Mn, Cu, Co, Se, Mg as the most abundant minerals; polyphenols.	Leaves	Methanol extract	Inductive paired plasma–optical emission spectrometer (ICP-OES) and phytochemical screening by *in vitro* assays	Antioxidant activity	[193]
*Dactylorhiza* *viridis* (L.) R.M. Bateman, Pridgeon and M.W. Chase	Quercetin, kaempferol.	Leaves	Methanol extract	2D-PC and UV spectral analysis		[169]
	Coelovirins A-G; 4-hydroxybenzaldehyde, 4-hydroxybenzyl alcohol, 4,4′-dihydroxydibenzyl ether, 4,4′-dihydroxydiphenylmethane, 4-(4-hydroxybenzyloxy) benzyl alcohol, gastrodin, quercetin-3,7-diglucoside, thymidine, loroglossin, militarine, dactylorhin A, dactylorhin B, *β*-sitosterol and daucosterol.	Tuber	Ethanol extract	Chemical and spectroscopic methods, including 2D-NMR techniques		[194]
	Dactylorhin B, loroglossin, dactylorhin A, militarine as the most abundant compounds.	Tuber	Ethanol extract	HPLC-DAD	*In vivo* antioxidant, anti-inflammatory and antiapoptotic activity; inhibition of glutamate-induced neurotoxicity	[195,196,197,198,199,200,201,202]
	Verbenone, caryophyllene, *β*-terpineol, and *δ*-cadinene are the most abundant compounds.	Inflorescence	Floral scent	HS-SPME-GC–MS analysis		[192]
*Epipactis atrorubens* (Hoffm.) Besser	Quercetin, kaempferol.	Leaves	Methanol extract	2D-PC and UV spectral analyses		[169]
	Chrysanthemin, mecocyanin, epipactin, cyanin.	Flowers		Chemical hydrolysis, absorption spectroscopy, chromatography		[177]
	Chrysanthemin, cyanin, seranin, ophrysanin, orchicyanin II, serapianin, orchicyanin I.	Flowers	Methanol extract	TLC, HPLC, and thin-layer electrophoresis		[168]
	Polyphenols, soluble thiols, free proline, ascorbate.	Leaves from plants belonging to serpentine dumps	Ethanol extract	Phytochemical screening by *in vitro* assays and total protein determination	Non-enzymatic antioxidants play a role in the adaptive potential in plants belonging to polluted sites	[203]
*Epipactis* *helleborine* (L.) Crantz.	Alkaloids.					[204]
	Quercetin.	Leaves	Methanol extract	2D-PC and UV spectral analyses		[169]
	Mecocyanin, chrysanthemin, epipactin, cyanin.	Flowers		Chemical hydrolysis, absorption spectroscopy, chromatography		[177]
	Mannose-specific lectins.	Leaves	Protein fraction	Mannose-Sepharose-4B-affinity chromatography	Antiviral activity against HIV-1, HIV-2, CMV, RSV, and influenza A; evaluation on MT-4, HEL, HeLa, and MDCK cell lines	[172]
	Monomeric and dimeric mannose-binding proteins; lectin.	Leaves	Protein fraction	Mannose-Sepharose-4B/affinity chromatography	Antiviral activity against HIV-1, HIV-2	[171]
	Vanillin, furfural, ethanol, eugenol, methoxyeugenol; 3-{2-{3-{3-(benzyloxy) propyl}-3-indol, 7,8-didehydro-4,5-epoxy-3,6-d-morphinan and oxycodone + other minor compounds such as alcohols and saturated hydrocarbons.	Flowers	Flowers nectar	GC-MS analysis	Vanillin, furfural, ethanol and eugenol play a role in pollinator attraction; morphinan/indole derivatives contribute to the sluggish effect on insects and in their disorientation	[205]
*Epipactis* *leptochila* (Godfery) Godfery	Quercetin.	Leaves	Methanol extract	2D-PC and UV spectral analyses		[169]
*Epipactis* *palustris* (L.) Crantz	Quercetin.	Leaves	Methanol extract	2D-PC and UV spectral analyses		[169]
	Epipactin, mecocyanin.	Flowers		Chemical hydrolysis, absorption spectroscopy, chromatography		[177]
*Epipactis veratrifolia* Boiss. and Hohen.	Quercetin.	Leaves	Methanol extract	2D-PC and UV spectral analyses		[169]
*Goodyera repens* (L.) R.Br.	Alkaloids.					[204]
	Kinsenoside, goodyeroside A, gastrodin.		Methanol extract	HPLC-MS/MS analysis		[206]
*Gymnadenia* *conopsea* (L.) R.Br.	Quercetin, kaempferol.	Leaves	Methanol extract	2D-PC and UV spectral analyses		[169]
	Orchicyanin I, orchicyanin II, cyanin.	Flowers		Chemical hydrolysis, absorption spectroscopy, chromatography		[177]
	Chrysanthemin, cyanin, seranin, ophrysanin, orchicyanin II, serapianin, orchicyanin I.	Flowers	Methanol extract	TLC, HPLC, and thin-layer electrophoresis		[168]
	Benzyl acetate; benzyl benzoate; methyl-eugenol; eugenol; alcohols, aldehydes, saturated hydrocarbons, terpenes.	Inflorescence	Flower scent	GC-MS and GC-EAD analysis	Benzyl acetate, eugenol, and benzyl benzoate are physiologically active on pollinators as assessed by electroantennography tests	[207]
	Phenylethylacetate, eugenol, 2-butenal, 1-butanol, hexanal, butyl acetate, methyl acetate as predominant compounds.	Inflorescence	Dynamic sampling of VOCs with custom packed glass multi-sorbent cartridge tubes	GC-MS analysis		[208]
*Gymnadenia* *lithopolitanica* (Ravnik) Teppner and E. Klein	Orchicyanin II, orchicyanin I, serapianin, cyanin, chrysanthemin, ophrysanin, seranin.	Flowers	Methanol extract	TLC, HPLC and thin-layer electrophoresis		[168]
*Gymnadenia* *nigra* (L.) Rchb. f.	Chrysanthemin.	Flowers		Chemical hydrolysis, absorption spectroscopy, chromatography		[177]
	Benzyl alcohol, 2-pehylethanol, phenylacetaldehyde, eicosanal, nonanal, benzaldehyde, vanillin; saturated hydrocarbons, alcohols, aldehydes, acids, terpenes.	Flowers	Volatile compounds isolated by steam distillation	GC-FID and GC-MS analyses		[209]
	Phenylacetaldehyde, tricosane, pentacosane, terpenes.	Stem and leaves	Volatile compounds isolated by steam distillation	GC-FID and GC-MS analyses		[209]
*Gymnadenia odoratissima* (L.) Rich.	Orchicyanin I, orchicyanin II, cyanin.	Flowers		Chemical hydrolysis, absorption spectroscopy, chromatography		[177]
	Phenylacetaldehyde, phenylethyl acetate, benzyl acetate, 1-phenyl-2,3-butandione, benzaldehyde, eugenol, (Z)-Isoeugenol/vanillin, phenylethyl alcohol, *α*-pinene, limonene; other compounds present in minor amounts (benzenoids; fatty acid derivatives, phenylpropanoids, isoprenoids).	Inflorescence	Floral scent	GC-MS and GC-EAD analyses	Benzaldehyde, phenylacetaldehyde, benzyl acetate, 1-phenyl-2,3-butandione, phenylethyl acetate, eugenol, and one unknown compound are physiologically active on pollinators as assessed by electroantennography	[207]
*Gymnadenia* *rubra* (Wettst.) K. Richt.	Orchicyanin II, ophrysanin, chrysanthemin, serapianin, orchicyanin I, seranin, cyanin.	Flowers	Methanol extract	TLC, HPLC, and thin-layer electrophoresis		[168]
*Gymnadenia spp. (G. corneliana* (Beauverd) Teppner and E.Klein; *G. nigra* (L.) Rchb. f.)	Chrysanthemin, ophrysanin, orchicyanin I, serapianin, orchicyanin II, seranin.	Flowers	Methanol extract	TLC, HPLC, and thin-layer electrophoresis		[168]
*Himantoglossum adriaticum* H. Baumann	Methyleugenol, 9-tricosene, tetradecane, pentadecane, and hexadecane as common compounds in plants sampled from different localities; oxalidone, pentadecyl hexanoate, *β*-ocimene were compounds varying in their relative amounts.	Inflorescence	Floral scent	HS-SPME-GC–MS analysis		[210]
*Himantoglossum caprinum* (M.Bieb.) Spreng.	Glucomannan in relatively high content.	Tuber	Tuber powder	FT-IR spectroscopy and enzymatic colorimetric method		[211]
*Himantoglossum hircinum* (L.) Spreng.	Orchinol, loroglossol, hircinol, *p*-hydroxybenzylalcohol.	Tuber			Orchinol and hircinol are active against the growth of *Candida lipolytica*	[212]
	Elemicin, eugenol, 3,4,5-trimethoxybenzaldehyde, benzyl benzoate, 3-(4,8,12-trimethyltridecyl) furan, 9-tricosene as predominant compounds.	Inflorescence	Floral scent	HS-SPME-GC–MS analysis		[210]
*Himantoglossum metlesicsianum* (W. P. Teschner) P. Delforge	Serapianin, orchicyanin I, seranin, orchicyanin II, chrysanthemin, cyanin, ophrysanin.	Flowers	Methanol extract	TLC, HPLC, and thin-layer electrophoresis		[168]
*Himantoglossum robertianum* (Loisel) P. Delforge	Orchicyanin II, cyanin, orchicyanin I, chrysanthemin.	Flowers		Chemical hydrolysis, absorption spectroscopy, chromatography		[177]
	Individuals from Greece: orchicyanin II, serapianin, seranin, cyanin, chrysanthemin, ophrysanin. Individuals from France: serapianin, orchicyanin I, orchicyanin II, seranin, ophrysanin, chrysanthemin, cyanin.	Flowers	Methanol extract	TLC, HPLC, and thin-layer electrophoresis		[168]
	Terpenoids (*α*-pinene, limonene, *β*-pinene), ethylidene-cyclopentane, acetone as predominant compounds.	Inflorescence	Dynamic sampling of VOCs with custom packed glass multi-sorbent cartridge tubes	GC-MS analysis		[208]
	High content in total phenols, flavonoids and proanthocyanidins; among compounds, the most abundant ones were scopoletin, kaempferol-3-O-rutinoside, caffeic acid, chlorogenic acid, epicatechin, roifolin, protocatecuic acid, vitexin, isovitexin, coumaric acid, catechin, apigenin.	Flowers	Hydroalcoholic extract	Phytochemical screening by *in vitro* assays and RP-HPLC-DAD analysis	Strong antioxidant activity both in cell-free and cell-based assays; inhibitory activity against skin matrix-degrading enzymes (elastase, collagenase) and *in vitro* wound-healing activity.	[213]
	Loroglossol, hircinol.	Tuber	Chloroform extract	H-NMR, ^13^C-NMR, 2D-NMR, and HPLC-MS	Antioxidant activity in polymorphonuclear leukocytes (PMN); antimicrobial properties against *Escherichia coli* and *Staphylococcus aureus*; anti-proliferative effect on gastric tumour cell lines by induction of apoptotic effect.	[17]
	Compounds present in variable content were mainly pristane, verbenone, *β*-sesquiphellandrene, caryophyllene, dihydrofarnesol, farnesol, *p*-menth-8-en-1-ol, *α*-farnesene, ethyl tetradecanoate, *α*-zingiberene, cis-*α*-bergamotene, *δ*-selinene, *β*-bisabolene, citronellol, methyl citronellate + other less abundant compounds.	Inflorescence	VOCs	HS-SPME-GC–MS analysis		[214]
*Limodorum abortivum* (L.) Sw.	Ophrysanin, chrysanthemin, seranin, orchicyanin I, serapianin, traces of cyanin and orchicyanin II.	Flowers	Methanol extract	TLC, HPLC, and thin-layer electrophoresis		[168]
*Neotinea tridentata* (Scop.) R.M. Bateman, Pridgeon and M.W. Chase	Orchicyanin I, serapianin, ophrysanin, orchicyanin II, seranin, cyanin, chrysanthemin.	Flowers	Methanol extract	TLC, HPLC, and thin-layer electrophoresis		[168]
*Neotinea ustulata* (L.) R.M. Bateman, Pridgeon and M.W. Chase	Mecocyanin, chrysanthemin.	Flowers		Chemical hydrolysis, absorption spectroscopy, chromatography		[177]
	Serapianin, seranin, ophrysanin, chrysanthemin.	Flowers	Methanol extract	TLC, HPLC, and thin-layer electrophoresis		[168]
	Quercetin.	Leaves	Methanol extract	2D-PC and UV spectral analyses		[169]
*Neottia ovata* (L.) Bluff and Fingerh.	Luteolin 3,4-diglucoside.	Leaves	Methanol extract	2D-PC and UV spectral analyses		[169]
	Chrysanthemin.	Flowers		Chemical hydrolysis, absorption spectroscopy, chromatography		[177]
	Sesquiterpenes; linalool; trans-*β*-ocimene; perillen; dendrolasin; hexadecanoic acid isopropyl ester.	Flowers	Floral scent	GC-MS analysis		[215]
	Mannose-specific lectins.	Leaves	Protein fraction	Mannose-Sepharose-4B/affinity chromatography; SDS-PAGE		[170]
	Mannose-binding isolectins.	Leaves	Protein fraction	Mannose-Sepharose-4B/affinity chromatography; SDS-PAGE	Agglutination activity on erythrocytes; antiretroviral activity against HIV-1/HIV-2	[171]
*Ophrys apifera* Huds.	Quercetin, kaempferol.	Leaves	Methanol extract	2D-PC and UV spectral analyses		[169]
	Chrysanthemin.	Flowers		Chemical hydrolysis, absorption spectroscopy, chromatography		[177]
	Kaempferol 3-O-*β*-D-glucoside, kaempferol 3-O-*β*-D-rutinoside, kaempferol 3-O-*β*-D-rhamnoside (high concentration in sepals; gymnostemium; traces found in the labellum).	Flowers	Defatted methanol extract	HPLC + spectroscopic techniques (UV–Vis and 1D and 2D NMR)		[216]
*Ophrys* *argolica s.l.* H.Fleischm.	Kaempferol 3-O-*β*-D-glucoside, kaempferol 3-O-*β*-D-rutinoside, kaempferol 3-O-*β*-D-rhamnoside (sepals, gymnostemium; traces found in the labellum).	Flowers	Defatted methanol extract	HPLC + spectroscopic techniques (UV–vis and 1D and 2D NMR)		[216]
*Ophrys* *bertolonii* Moretti	Terpenes (mainly caryophyllene, sesquiphellandrene, *α*-cyclocitral, 4-terpineol, *α*-pinene, copaene, 4-thujanol); aldehydes (mainly dodecanal, decanal, nonanal, octanal); 4-methyl-tetradecane; ketones (mainly 3.5-octadien-2-one, 2-nonen-4-one, pentadecanone); alcohols (mainly 3-hexen-1-ol, 2-nonanol); 4-methylphenol; 3-hexen-1-ol-acetate; dodecane; pentadecane; nonanoic acid.	Inflorescence of subsp. *benacensis*	Floral scent	HS-SPME-GC-MS analysis		[217]
*Ophrys bombyliflora* Link.	Quercetin, kaempferol.	Leaves	Methanol extract	2D-PC and UV spectral analyses		[169]
*Ophrys holosericea s.l.* (Burm.f.) Greuter	Ophrysanin, orchicyanin II, serapianin, orchicyanin I, chrysanthemin, seranin (*O. holosericea*); ophrysanin, chrysanthemin, serapianin, seranin (*O. holosericea* subsp. *lacaitae*).	Flowers	Methanol extract	TLC, HPLC and thin-layer electrophoresis		[168]
	Saturated hydrocarbons (mainly tricosane, pentacosane, heneicosane, heptacosane); unsaturated hydrocarbons (mainly -pentacosene, 9-tricosene, 9-pentacosene, 7-tricosene, 7-heptacosene, 9-heptacosene, 11-pentacosene); aldehydes (nonanal, phenylacetaldehyde, heptanal, octadecanal); benzil alcohol + other minor compounds including terpenes.	Inflorescence	EOs isolated by steam distillation	GC-FID and GC-MS analyses	Antioxidant activity (DPPH assay)	[175]
*Ophrys* *insectifera s.l.* L.	Chrysanthemin.	Flowers		Chemical hydrolysis, absorption spectroscopy, chromatography		[177]
	Chrysanthemin, ophrysanin, serapianin, seranin + traces of orchicyanin I, cyanin, orchicyanin II.	Flowers	Methanol extract	TLC, HPLC, and thin-layer electrophoresis		[168]
*Ophrys* *ferrum-equinum* Desf.	Chrysanthemin, ophrysanin, seranin, serapianin.	Flowers	Methanol extract	TLC, HPLC, and thin-layer electrophoresis		[168]
*Ophrys fusca s.l.* Link.	Chrysanthemin, seranin, ophrysanin, serapianin.	Flowers	Methanol extract	TLC, HPLC, and thin-layer electrophoresis		[168]
*Ophrys lutea s.l.* Cav.	Ophrysanin, chrysanthemin, serapianin, seranin (*O. lutea* subsp. *galilaea*).	Flowers	Methanol extract	TLC, HPLC, and thin-layer electrophoresis		[168]
*Ophrys reinholdii* Spruner ex Fleischm.	Ophrysanin, orchicyanin I, chrysanthemin (*O. reinholdii* subsp. *straussii*).	Flowers	Methanol extract	TLC, HPLC, and thin-layer electrophoresis		[168]
*Ophrys scolopax s.l.* Cav.	Chrysanthemin, seranin, serapianin, ophrysanin (*O. scolopax* subsp. *cornuta*); orchicyanin I, ophrysanin, serapianin, chrysanthemin (*O*. *scolopax* subsp. *phrygia*).	Flowers	Methanol extract	TLC, HPLC, and thin-layer electrophoresis		[168]
	Kaempferol 3-O-*β*-D-glucoside, kaempferol 3-O-*β*-D-rutinoside, kaempferol 3-O-*β*-D-rhamnoside (sepals, gymnostemium; traces found in the labellum).	Flowers from subsp. *cornuta*	Defatted methanol extract	HPLC + spectroscopic techniques (UV–vis and 1D and 2D NMR)		[216]
*Ophrys speculum* Link.	Chrysanthemin, orchicyanin I, orchicyanin II.	Flowers		Chemical hydrolysis, absorption spectroscopy, chromatography		[177]
	Ophrysanin, chrysanthemin, serapianin, seranin.	Flowers	Methanol extract	TLC, HPLC, and thin-layer electrophoresis		[168]
	Cyanidin pigments in the labellar speculum; flavonoids in the brown labellar margins (delphinidin, quercetin).	Flowers	0.1% HCl/methanol extract	Spectrophotometric analyses and HPLC-DAD-TOF/MS		[218]
*Ophrys* *sphegodes s.l.* Mill.	Ophrysanin, chrysanthemin, seranin, serapianin, orchicyanin I.	Flowers	Methanol extract	TLC, HPLC, and thin-layer electrophoresis		[168]
	Terpenes (mainly D-limonene, *α*-zingibirene, *α*-pinene, cyclosativene, *β*-pinene, 4-terpineol, menthol, isolongifolene, eucarvone, *o*-cymene, *β*-myrcene, naphtalene); undecane; formic acid;alcohols (mainly heptanol, 3-decen-1-ol); 3-hexen-1-ol-acetate; 2-nonanal; phenols (mainly phenol, 4-methylanisole); dimethyl sulfone.	Inflorescence	Floral scent	HS-SPME-GC-MS analysis		[217]
*Ophrys* *tenthredinifera* Willd.	Quercetin, kaempferol.	Leaves	Methanol extract	2D-PC and UV spectral analyses		[169]
	Ophrysanin, orchicyanin II, serapianin, chrysanthemin, cyanin.	Flowers	Methanol extract	TLC, HPLC, and thin-layer electrophoresis		[168]
*Orchis anatolica* Boiss.	9,10-Dihydrophenanthrenes (orchinol and hircinol).	Tuber	Enzyme preparations from induced orchid bulbs to accumulate phytoalexins	2D-PC and TLC		[178]
	Orchicyanin I + unknown anthocyanins.	Flowers	Methanol extract	TLC, HPLC, and thin-layer electrophoresis		[168]
	Flavonoids, triallate, theo-bromade, tannins.	Tuber	Hydroalcoholic extract characterized in previous research		Antidiabetic/antihyperglycemic effect: reduction of blood sugar values, but the extract did not bring to normal blood value levels	[219]
*Orchis* *anthropophora* (L.) All.	Quercetin, kaempferol.	Leaves	Methanol extract	2D-PC and UV spectral analyses		[169]
		Roots, stem, inflorescence	Ethanol extracts from plants sampled in Italy		The extracts showed negative results in inhibiting the growth and biofilms of methicillin-resistant *Staphylococcus aureus*	[220]
	Sesquiterpenes (*β*-caryophyllene; caryophylladienol); monoterpenes (terpenoid, 1,8 cyneole, limonene, *α*-pinene, *β*-pinene, myrcene, eucalyptol); fatty acid derivatives; nonanal; undecane; alkanoid; benzene acetaldehyde; methyl salicilate; ethylacetophenone.	Inflorescence	Floral scent collected from adult plants in natural population	GC-MS analysis		[221]
*Orchis italica* Poir.	Quercetin.	Leaves	Methanol extract	2D-PC and UV spectral analyses		[169]
	9,10-Dihydrophenanthrenes (orchinol and hircinol).	Tuber	Enzyme preparations from induced orchid bulbs to accumulate phytoalexins	2D-PC and TLC		[178]
		Stem, leaves, inflorescence	Ethanol extracts from plants sampled in Italy		The extracts showed negative results in inhibiting the growth and biofilms of methicillin-resistant *Staphylococcus aureus*	[220]
	Caryophyllene, 4-(3-hydroxy-2-methoxyphenyl) butan-2-one, eucalyptol, heptadecane, heinecosane, methyl 3,5-dimethoxybenzoate, ethyl dodecanoate, hexadecane, pentadecane, isopropyl palmitate, nonadecane, tricosane, octadecane.	Inflorescence	Floral scent collected with HS-SPME from adult plants (Southern Italy)	GC–MS analysis		[222]
*Orchis mascula s.l.* (L.) L.	Quercetin, kaempferol.	Leaves	Methanol extract	2D-PC and UV spectral analyses		[169]
	Orchicyanin I, orchicyanin II, cyanin.	Flowers		Chemical hydrolysis, absorption spectroscopy, chromatography		[177]
	9,10-Dihydrophenanthrenes (orchinol and hircinol).	Tuber	Enzyme preparations from induced orchid bulbs to accumulate phytoalexins	2D-PC and TLC		[178]
	*trans-*β-ocimene; tricyclene; α-pinene; linalool + less abundant sesquiterpene compounds.	Inflorescence	Floral scent	GC-MS analysis		[223]
	Ophrysanin, cyanin, orchicyanin I, seranin, serapianin, orchicyanin II, chrysanthemin (*O. mascula s.l.*); orchicyanin II, serapianin, seranin, orchicyanin I, ophrysanin (*O. mascula* subsp. *olbiensis*).	Flowers	Methanol extract	TLC, HPLC, and thin-layer electrophoresis		[168]
	Isoprenoids (*E*-ocimene/styrene; linalool; 1.8-cineole; limonene; sabinene; myrcene; cis-linalool oxide; *β*-farnesene; (Z)-ocimene; *α*-terpineol); 2-methyl-6-methylen-3,7-octadien-2-ol/acetophenone; nonanal; benzaldehyde.	Flowers	Floral scent	GC-MS analysis		[224]
	Ocimene-(E); linalool; limonene; (Z)-3-hexenyl acetate linalool oxide trans; 1.8-cineole; myrcene; sabinene; allo-ocimene-(Z); 6-methylhepten-2-one; ocimene-(Z); *β*-pinene; methyl dodecanoate; (Z)-3-hexenyl butyrate; *β*-farnesene; α-terpineol.	Inflorescence	Floral scent	HS-SPME-GC-MS		[225]
	Loroglossin, *p*-hydroxybenzylalcohol, orchinol; high content of mucilage.	Tuber				[13]
	Linalool; eucalyptol; 6,10,14-trimethyl-2-pentadecanone; pentadecane; tetradecane; hexadecane; tridecane; heptadecane; pristane; limonene; octadecane.	Inflorescence	Floral scent	HS-SPME-GC–MS analysis		[222]
	Saponins; flavonoids; anthraquinone; terpenoids; tannins; cyanogenic glycosides; cardiac glycosides; 2-methyl-Z,Z-3,13-octadecadienol, n-hexadecanoic acid, 2 furancarboxaldehyde 5-(hydroxymethyl), 2-propanone, 1,1-diethoxy-, D-allose, 1,6-anhydro-à-D-galactofuranose, 3-acetylthymine, DL-4-amino-3-hydroxybutyric acid.	Flowers	Ethanol extract	Phytochemical screening, FT-IR/GC MS analyses	Antibacterial activity against *Salmonella paratyphi, Salmonella typhi, S. paratyphi, Staphylococcus aureus, Vibrio cholerae, Klebsiella oxytoca, Escherichia coli*, and *Vibrio parahaemolyticus*	[226]
*Orchis* *militaris* L.	*p*-hydroxybenzylalcohol and orchinol are produced when the plant is challenged with *Rhizoctonia repens*.	Tuber				[227]
	Loroglossin, militarine.	Whole plant	Polarity-based sequential extraction	Preparative chromatography; UV-spectroscopy; NMR, IR		[228]
	Cyanin, orchicyanin II, orchicyanin I.	Flowers		Chemical hydrolysis, absorption spectroscopy, chromatography		[177]
	Coumarin, 4-hydroxy benzyl methyl ether, isoquercitrin, astragalin, melilotoside, militarine.	Whole plant	Polarity-based sequential extraction	Droplet counter-current chromatography (DCCC), UV-spectroscopy,1H-NMR, ^13^C-NMR and FABMS analyses		[229]
	Cyanin, orchicyanin II, orchicyanin I, chrysanthemin, ophrysanin, seranin, cyanin.	Flowers	Methanol extract	TLC, HPLC, and thin-layer electrophoresis		[168]
*Orchis* *pallens* L.	*β*-curcumene; *α*-zingiberene, diethyltoluamide, di-epi-*α*-cedrene; pentadecane; *β*-farnesene; hexadecane; heptadecane; caryophyllene; tetradecane; 6,10,14-trimethyl-2-pentadecanone; pristane; octadecane.	Inflorescence	Floral scent	HS-SPME-GC–MS analysis		[222]
*Orchis* *patens* Desf.	Orchicyanin I, orchicyanin II, ophrysanin, cyanin, serapianin, chrysanthemin.	Flowers	Methanol extract	TLC, HPLC, and thin-layer electrophoresis		[168]
*Orchis* *pauciflora* Ten.	Isoprenoids (myrcene, (E)-ocimene/styrene, ipsdienol, *β*-farnesene, 6-methyl-5-hepten-2one, limonene, *α*-terpineol, linalool); 2-methyl-6-methylen-3,7-octadien-2-ol/acetophenone; nonanal; benzaldehyde; hydroquinone dimethyl ether; 2-methyl-6-methylene-1,3,7-octatriene.	Flowers	Floral scent	GC-MS analysis		[224]
	(E)-*β*-farnesene; (E,E)-*α*-farnesene; limonene/1,8-cineol; acetophenone + other less abundant compounds.	Inflorescence	Floral scent	HS-SPME-GC–MS analysis	(E)-*β*-farnesene elicited the physiological response in *Bombus terrestris* and significantly increased the pollinia export	[230]
	Linalool; 1,4-dimethoxybenzene; germacrene D; 6,10,14-trimethyl-2-pentadecanone; pentadecane; 1,2,4-trimethoxybenzene; tetradecane; eucalyptol; hexadecane; ethyldodecanoate; *β*-farnesene; Epi-bicyclosesquiphllandrene; heptadecane; tridecane + other less abundant compounds.	Inflorescence	Floral scent	HS-SPME-GC–MS analysis		[222]
*Orchis* *provincialis* Balb. ex Lam. and DC.	β-farnesene; farnesal; 6,10,14-trimethyl-2-pentadecanone; heptadecane; limonene; α-terpineol + other less abundant compounds.	Inflorescence	Floral scent	HS-SPME-GC–MS analysis		[222]
*Orchis* *purpurea* Huds.	Orchicyanin II, cyanin, orchicyanin I, chrysanthemin.	Flowers		Chemical hydrolysis, absorption spectroscopy, chromatography		[177]
	Orchicyanin I, cyanin, chrysanthemin, seranin, ophrysanin, serapianin, orchicyanin II.	Flowers	Methanol extract	TLC, HPLC, and thin-layer electrophoresis		[168]
		Stem, leaves and inflorescence	Ethanol extracts		No inhibition of the growth and biofilm formation of methicillin-resistant *Staphylococcus aureus* was observed	[220]
	Coumarin; *p*-cresol; pentacosane; hexadecanoic acid; *p*-vinyl-phenol and other less abundant compounds.	Inflorescence	EOs isolated by steam distillation	GC-MS analyses		[231]
*Orchis simia* Lam.	Orchicyanin II, orchicyanin I, cyanin.	Flowers		Chemical hydrolysis, absorption spectroscopy, chromatography		[177]
	*α*-pinene; eucalyptol; linalool; ethylacetophenone; *β*-pinene; myrcene; alkanoid; sabinene; benzene acetaldehyde; *α*-campholenal; linalool oxide; methyldodecane; *cis*-hydrate sabinene; methylsalicilate; *β*-caryophyllene; 1,8 cineol; undecane; limonene + other less abundant compounds.	Inflorescence	Floral scent	GC-MS analysis		[221]
	*α*-pinene; *β*-phellandrene; myrcene; sabinene; *β*-pinene; nonanal; terpinene-4-ol; camphenol-6 al; limonene; *γ*-terpinene; sabinene hydrate trans; (Z)-3-hexenyl acetate; terpinolene; decanal; pinocarvone; *β*-caryophyllene; *α*-terpineol; *α*-campholenal + other less abundant compounds.	Inflorescence	Floral scent	GC-MS analysis		[225]
*Orchis spitzelii* Saut. ex W.D.J. Koch	Serapianin, seranin, orchicyanin I (plants growing in France); orchicyanin I, cyanin, seranin, orchicyanin II, serapianin (plants growing in Greece).	Flowers	Methanol extract	TLC, HPLC, and thin-layer electrophoresis		[168]
*Platanthera bifolia* (L.) Rich.	Quercetin, kaempferol, 6-hydroxy-C-glycosides.	Leaves	Methanol extract	2D-PC and UV spectral analyses		[169]
	*trans*-*β*-ocimene; lilac aldehydes; methyl benzoate; lilac alcohols; geraniol; benzyl benzoate; benzyl alcohol in variable content depending on the presence of pollinated or unpollinated flowers; linalool; other less abundant compounds	Inflorescence	Floral scent	GC-MS analysis		[232]
	Flowers from different populations showed a variable content in Linaloolic compounds (furanoids, l-(S-ethenyl-5-methyl tetrahydrofuran-2-yl) ethanal, linalool, 2-hydroxy-S-ethenyl-5-methyl tetrahydrofuran, 5-ethenyl-5-methyl-dihydro-2(3H)-furanone); lilac compounds (lilac alcohols, lilac aldehydes, lilac alcohol acetate); isoprenoids (trans-*β*-ocimene, 6-methyl-5-hepten-2-one, *p*-cymen-8-ol); geraniolic compounds (neral, geranial, citronellol, geraniol, nerol); benzenoids (*α*-*p*-dimethylstyrene. benzaldehyde, methyl benzoate, benzyl acetate, methyl salicylate, benzyl alcohol, cinnamic aldehyde, cinnamyl acetate, eugenol, cinnamyl alcohol, isoeugenol, benzyl benzoate).	Flowers	Floral scent	GC-MS analysis		[233]
	Methyl benzoate, benzyl benzoate, benzyl salicylate, methyl salicylate, cinnamyl alcohol, lilac aldehydes, hexadecanoic acid methyl ester, benzaldehyde + other compounds.	Inflorescence	Floral scent dichloromethane/ethanol extract	GC-EAD and GC-MS analyses	Benzyl benzoate, benzyl salicylate, cinnamyl alcohol, methyl benzoate, methyl salicylate and especially lilac aldehydes were electrophysiologically active on *Autographa gamma*	[234,235]
	Flowers of subsp. *osca* were collected in different populations of Southern Italy, showing: benzyl benzoate, linalool, benzyl 2-hydroxybenzoate, methyl benzoate, *β*-ocimene (Grisolia); lilac aldehydes, lilac alcohols, heptadecane, hexadecane, pentadecane, caryophyllene + other less abundant compounds (Pignola); lilac aldehydes, lilac alcohols, linalool, pentadecane, hexadecane + other minor compounds (Marsico Nuovo); benzyl benzoate, methyl benzoate, geraniol, benzyl 2-hydroxybenzoate, citral, octadecane (Palena).	Flowers	Floral scent	HS-SPME-GC-MS analysis		[236]
	Phenolic compounds and flavonoids detected in higher quantity in individuals from disturbed habitats in respect to those from natural sites.	Leaves	Hydroalcoholic extract	Phytochemical screening by *in vitro* assays		[237]
*Platanthera chlorantha* (Custer) Rchb.	Different amounts of the same compounds reported for *P. bifolia* (see above).	Inflorescence	Floral scent	GC-MS analysis		[233]
	Lilac alcohol D, lilac aldehyde A, lilac aldehyde B, lilac aldehyde C, germacrene D + other minor compounds.	Inflorescence	Floral scent	HS-SPME-GC-MS analysis		[236]
	Lilac aldehyde, 3,7-dimethyl-1,3,6-octatriene, 3-carene, lilac alcohol, 3,7-dimethyl-2,6-octadien-1-ol acetate + benzyl acetate in minor amounts.	Inflorescence	Floral scent hexane extract	GC-MS analysis		[238]
*Pseudorchis* *albida* Ά. Löve and D. Löve	Quercetin, kaempferol.	Leaves	Methanol extract	2D-PC and UV spectral analyses		[169]
	Floral scent analysed in two different populations showing variable content of: limonene, *β*-myrcene, (Z)-verbenol, verbenone, 4-oxoisophorone, *β*-phellandrene, (E)-sabinene hydrate, (Z)-sabinene hydrate, *β*-sabinene, 2-hydroxypinane-3-one + other minor compounds (flowers).	Inflorescence		DHA-GC-MS analysis		[239]
*Serapias cordigera* L.	Quercetin, kaempferol.	Leaves	Methanol extract	2D-PC and UV spectral analyses		[169]
	Individuals from France, Italy or Greece showed variable content in serapianin, seranin, chrysanthemin, ophrysanin, orchicyanin II, cyanin, orchicyanin I.	Flowers	Methanol extract	TLC, HPLC, and thin-layer electrophoresis		[168]
*Serapias lingua* L.	Quercetin.	Leaves	Methanol extract	2D-PC and UV spectral analyses		[169]
	Orchicyanin II, orchicyanin I, mecocyanin, chrysanthemin, epipactin.	Flowers		Chemical hydrolysis, absorption spectroscopy, chromatography		[177]
	Individuals from France or Greece showed variable content in serapianin, seranin, chrysanthemin, ophrysanin, orchicyanin II, orchicyanin I, cyanin.	Flowers	Methanol extract	TLC, HPLC, and thin-layer electrophoresis		[168]
*Serapias neglecta* De Not.	Serapianin, seranin, ophrysanin, orchicyanin I, orchicyanin II, chrysanthemin.	Flowers	Methanol extract	TLC, HPLC, and thin-layer electrophoresis		[168]
*Serapias nurrica* Corrias.	Serapianin, seranin, ophrysanin, orchicyanin II, chrysanthemin, orchicyanin I.	Flowers	Methanol extract	TLC, HPLC, and thin-layer electrophoresis		[168]
*Serapias olbia* Verg.	Ophrysanin, serapianin, seranin, orchicyanin II, orchicyanin I, chrysanthemin.	Flowers	Methanol extract	TLC, HPLC, and thin-layer electrophoresis		[168]
*Serapias* *parviflora* Parl.	Glucomannan in relatively high content.	Tuber	Tuber powder	FT-IR spectroscopy and enzymatic colorimetric methods		[211]
*Serapias* *vomeracea* (Burm. f.) Briq.	Quercetin, kaempferol.	Leaves	Methanol extract	2D-PC and UV spectral analyses		[169]
	Glucomannan in relatively high content.	Tuber	Tuber powder	FT-IR spectroscopy and enzymatic colorimetric methods		[211]
	Serapianin, seranin, ophrysanin, orchicyanin II, orchicyanin I, chrysanthemin.	Flowers	Methanol extract	TLC, HPLC, and thin-layer electrophoresis		[168]
*Spiranthes aestivalis* (Poir.) Rich.	Quercetin, kaempferol.	Leaves	Methanol extract	2D-PC and UV spectral analyses		[169]
*Traunsteinera globosa* (L.) Rchb.	Orchicyanin II, orchicyanin I, cyanin, seranin, serapianin, chrysanthemin, ophrysanin.	Flowers	Methanol extract	TLC, HPLC, and thin-layer electrophoresis		[168]

## Data Availability

Not applicable.
